# Spatial Learning Promotes Adult Neurogenesis in Specific Regions of the Zebrafish Pallium

**DOI:** 10.3389/fcell.2022.840964

**Published:** 2022-05-11

**Authors:** Laura S. Mazzitelli-Fuentes, Fernanda R. Román, Julio R. Castillo Elías, Emilia B. Deleglise, Lucas A. Mongiat

**Affiliations:** ^1^ Departamento de Física Médica, Centro Atómico Bariloche, Comisión Nacional de Energía Atómica, San Carlos de Bariloche, Argentina; ^2^ Consejo Nacional de Investigaciones Científicas y, Técnicas, Argentina; ^3^ Instituto Balseiro, Centro Atómico Bariloche, San Carlos de Bariloche, Argentina; ^4^ Centro Regional Universitario Bariloche, Universidad Nacional del Comahue, San Carlos de Bariloche, Argentina; ^5^ Facultad de Ciencias Exactas y Naturales, Universidad de Buenos Aires, Ciudad Autónoma de Buenos Aires, Argentina

**Keywords:** neural stem/progenitor cells, plasticity, telencephalon, danio rerio (zebrafish), spatial learning and memory

## Abstract

Adult neurogenesis could be considered as a homeostatic mechanism that accompanies the continuous growth of teleost fish. As an alternative but not excluding hypothesis, adult neurogenesis would provide a form of plasticity necessary to adapt the brain to environmental challenges. The zebrafish pallium is a brain structure involved in the processing of various cognitive functions and exhibits extended neurogenic niches throughout the periventricular zone. The involvement of neuronal addition as a learning-related plastic mechanism has not been explored in this model, yet. In this work, we trained adult zebrafish in a spatial behavioral paradigm and evaluated the neurogenic dynamics in different pallial niches. We found that adult zebrafish improved their performance in a cue-guided rhomboid maze throughout five daily sessions, being the fish able to relearn the task after a rule change. This cognitive activity increased cell proliferation exclusively in two pallial regions: the caudal lateral pallium (cLP) and the rostral medial pallium (rMP). To assessed whether learning impinges on pallial adult neurogenesis, mitotic cells were labeled by BrdU administration, and then fish were trained at different periods of adult-born neuron maturation. Our results indicate that adult-born neurons are being produced on demand in rMP and cLP during the learning process, but with distinct critical periods among these regions. Next, we evaluated the time course of adult neurogenesis by pulse and chase experiments. We found that labeled cells decreased between 4 and 32 dpl in both learning-sensitive regions, whereas a fraction of them continues proliferating over time. By modeling the population dynamics of neural stem cells (NSC), we propose that learning increases adult neurogenesis by two mechanisms: driving a chained proliferation of labeled NSC and rescuing newborn neurons from death. Our findings highlight adult neurogenesis as a conserved source of brain plasticity and shed light on a rostro-caudal specialization of pallial neurogenic niches in adult zebrafish.

## Introduction

Brain plasticity allows neuronal circuits to adapt to environmental demands, promoting the ability to cope with the surrounding world. Adult neurogenesis, the generation and integration of new neurons in the brain of adult organisms, constitute a major source of brain plasticity (Mongiat and Schinder, 2011; Sailor et al., 2017; Toda and Gage, 2018)⁠. The occurrence of neurogenesis in adult brains extends throughout the vertebrate subphylum, although its magnitude varies greatly along phylogeny (Kaslin et al., 2008; Grandel and Brand, 2013; Alunni and Bally-Cuif, 2016; Zupanc, 2021)⁠. In mammals, adult neurogenesis is restricted mostly to the hippocampal dentate gyrus and the olfactory bulb (Lledo et al., 2006)⁠, whereas in teleost fish adult-born neurons are generated throughout their brain (Kaslin et al., 2008)⁠.

The zebrafish (*Danio rerio*) possesses a great neurogenic potential evidenced by constitutive neural stem cell (NSC) proliferation in almost all subdivisions of their brain, generating a variety of adult-born neurons (Zupanc et al., 2005; Adolf et al., 2006; Grandel et al., 2006)⁠. One of the main brain regions in which adult neurogenesis has been studied in zebrafish is the dorsal telencephalon, or pallium. In contrast to other vertebrates, the telencephalon of ray-finned fish develops by an eversion of the neural tube (Folgueira et al., 2012)⁠. In consequence, both pallial hemispheres are separated and enclosed by a T-shaped ventricle. In this structure, the neuronal progenitors are localized in the periventricular zone (Adolf et al., 2006; Grandel et al., 2006)⁠. In the zebrafish pallium, there are different kinds of neural progenitor subtypes. Some of them, notably radial glia (RG) of the pallium, are considered NSCs, resembling the pallial RG in the mouse adult neurogenic niches (Than-Trong and Bally-cuif, 2015)⁠. These NSCs can be classified according to their proliferation activity, such as quiescent and active NSCs. At any time, the active NSCs correspond to ∼5% of total RG in the periventricular zone (März et al., 2010)⁠. In turn, the active NSCs can suffer symmetric or asymmetric divisions to maintain the NSC reservoir and give rise to progeny with neurogenic potential (Than-Trong et al., 2020)⁠. Moreover, there is a different subset of neuronal progenitors in the pallial periventricular zone. These cells are negative for astroglial markers and exhibit intense mitotic activity with neurogenic commitment (Rothenaigner et al., 2011; Than-Trong et al., 2020)⁠. Previous studies proposed that this neuronal progenitor population resembles the Transit Amplifying Progenitors described in rodents (März et al., 2010)⁠.

After adopting neuronal phenotype, the new neurons migrate radially a few micrometers into the pallial parenchyma and become integrated into the adult pallial networks. This neurogenic process leads to an outside-in architecture, where neurons generated in early development are positioned at the center of the pallium, while a layered gradient of newer neurons is distributed towards the periphery (Furlan et al., 2017)⁠. The new neurons mature and integrate into the pallial neuronal networks (Grandel et al., 2006; Rothenaigner et al., 2011; Lange et al., 2020; Than-Trong et al., 2020)⁠. Recently, Lange and coworkers performed single-cell sequencing of NSC’s progeny to characterize the intrinsic heterogeneity of adult-born neurons in the zebrafish telencephalon, revealing a striking homology with the neuronal types found in the mammalian forebrain (Lange et al., 2020)⁠. Therefore, adult neurogenesis contributes to a continuous rearrangement of zebrafish’s pallial networks.

Several studies have been conducted to decipher the organization of the teleost pallium based on anatomical characterization and the expression of molecular markers (Wullimann and Mueller, 2004; Vargas et al., 2009; Mueller, 2011; Harvey-Girard et al., 2012; Ganz et al., 2015)⁠. Current knowledge on the functional role of the different pallial regions, is complemented with a series of functional studies involving distinct learning paradigms in combination with the detection of neuronal activity (by different proxies) and/or lesions on specific regions (Portavella et al., 2004; Durán et al., 2010; Trotha et al., 2014; Elliott et al., 2017; Lal et al., 2018; Ausas et al., 2019)⁠. All these works indicate that the teleost dorsomedial telencephalic region (medial pallium, MP) is homologus to the basolateral amygdala of mammals, whereas the dorsolateral telencephalic region (lateral pallium, LP) is homologus to the mammalian hippocampus. From these studies emerge a strong relation between the teleost pallium and cognitive activity, suggesting plastic changes on pallial networks. In particular, adult neurogenesis could play a relevant role by shaping the neuronal circuits during learning. In rodents, it is known that behavioral paradigms such as odor-related and hippocampus-dependent learning increase the survival of immature adult-born neurons (Lledo et al., 2006; Anderson et al., 2011; Aasebø et al., 2018)⁠. However, to our knowledge, the role of cognitive activity on pallial network remodeling has not been explored in teleosts yet.

In this study, we trained adult zebrafish in a cognitive task to evaluate the impact of sustained neuronal activity on pallial adult neurogenesis. We found that learning may lead to plastic network remodeling driven by adult neurogenesis, specifically in the rostral MP and caudal LP regions.

## Results

### Adult Zebrafish Learn in the Cue-Guided Rhomboid Maze

**FIGURE 1:**
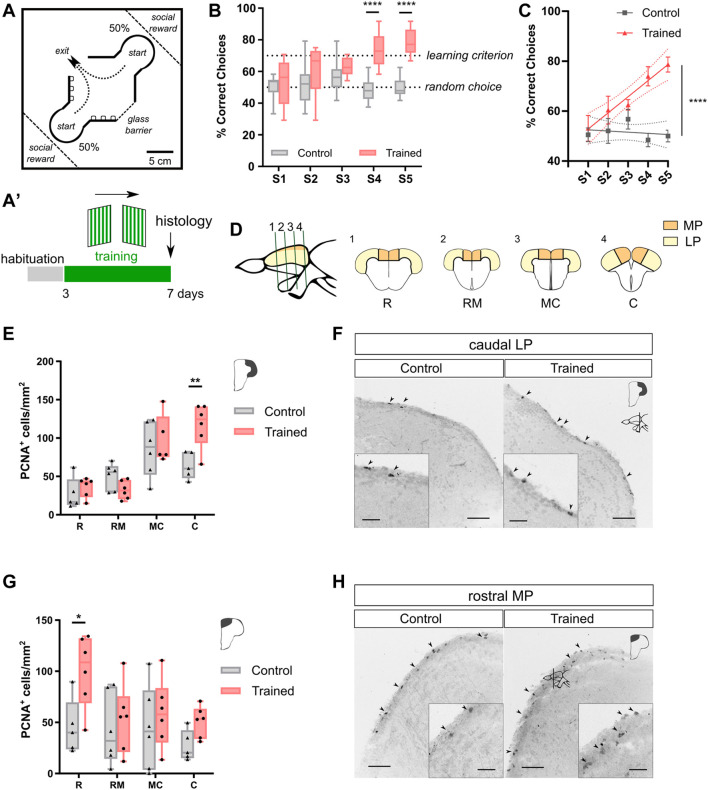
Training adult zebrafish in a rhomboid maze boosts cell proliferation in the rMP and the cLP. **(A)**. Experimental device. The maze contains two starting boxes, and two possible exits, one of which is blocked with a glass barrier. Fish were trained to find the correct exit, orientating themselves with cues placed on two walls. On the edges of the tank, two glass enclosures contained fish’s conspecifics as social reward. On each trial, fish were placed in a start box, and, once they reached the central arena, a correct choice was scored if they swam through the exit, and a failure if they bumped against the glass barrier. Each daily session consisted of 24 trials. **(A')** Behavioral schedule. Cue patterns and exit position (arrow) are specified above. Fish were habituated to the experimental tank for 2 days, and subsequently trained for five consecutive days. **(B)**. Learning curves for Trained and Control individuals. Trained fish reach the learning criterion after five consecutive sessions. Controls do not exhibit a learning curve (Two-way RM ANOVA, Treatment effect: F_(1, 14)_ = 25.78 with *p* = 0.0002, Session effect: F_(4, 56)_ = 3.422 with *p* = 0.0143. Bonferroni’s multiple comparisons test, **** depicts *p* < 0.0001. Trained, N = 8; Control N = 8). Dashed line at 70% correct choices depicts learning criterion; dashed line at 50% correct choices indicates random choice. **(C)**. Simple linear regression for Trained and Control individuals (ANCOVA, F_(1, 77)_ = 9.010. **** depicts *p* < 0.0001. Trained, N = 8; Control N = 8). Dashed line indicates 95% confidence intervals. **(D)**. Left: Sagittal schematic view of zebrafish forebrain, indicating the position of the cross sections on the right. Right: Cross sections of zebrafish telencephalon along rostro-caudal axis (R: rostral, RM: rostro-medial, MC: medio-caudal, C: caudal). The colored regions depict MP and LP. **(E)**. PCNA^+^ cells in LP. (Two-way ANOVA, Treatment effect: F_(1, 37)_ = 3.873 with *p* = 0.0566, Pallium region effect: F_(3, 37)_ = 20.27 with *p* < 0.0001. Bonferroni’s multiple comparisons test, ** denotes *p* < 0.001. Trained, N = 6; Control, N = 6). **(F)**. Cross sections of telencephalic pallium immunostained for PCNA in cLP of Trained and Control individuals. Scale bar, 50 μm. Scale bar in higher magnifications, 20 μm. Black arrows indicate representative PCNA^+^ cells. **(G)**. PCNA^+^ cells in MP. (Two-way ANOVA, Treatment effect: F_(1, 38)_ = 7.796 with *p* = 0.0082, Pallium region effect: F_(3, 38)_ = 2.332 with *p* = 0.0895. Bonferroni’s multiple comparisons test, * denotes *p* < 0.05. Trained, N = 6; Control, N = 6). **(H)**. Cross sections of telencephalic pallium immunostained for PCNA in rMP of Trained and Control individuals. Scale bar, 50 μm. Scale bar in higher magnifications, 20 μm. Black arrows indicate representative PCNA^+^ cells.

We hypothesized that sustained pallial neuronal activity, triggered as a consequence of a learning routine, would enhance the rate of adult neurogenesis in the circuits underlying the cognitive function. For this purpose, we looked for a learning paradigm involving the function of specific pallial structures, together with a sustained cognitive challenge. We adapted a cue-guided rhomboid maze, developed for goldfish, in order to train adult zebrafish in learning a spatial task by relating external cues with their internal-directional information. Otherwise noticed, 10 ± 1 months-old adult AB-wild type zebrafish (*Danio rerio*) were used throughout this work. During training, fish were located randomly at one of the two possible start sites in each trial. The fish had to learn the relationship between the cues’ position and the exit to solve the maze; for example, turn right if cues are located at the same side of the start compartment and turn left if they are at the opposite side (Figure 1A). Thus, on each trial the fish must take a left-right decision based on cues’ position. To encourage the completion of the task, we placed several conspecifics in outer compartments as social reward. To assess the ability of zebrafish to learn the task, fish were trained for five consecutive sessions (1 session/day, 24 trials/session, Figure 1A’). The Control group consisted on fish which were subjected to the same training routine, with the exception that the glass barrier was randomly placed in any of the exits to avoid learning. The Control group exhibited a ∼50% correct choices throughout the sessions, remaining close to the chance level (Figure 1B,C, Supplementary Video S1). In contrast, the Trained group increased their performance in a daily manner until they exceeded a 75% of correct choices (Percentage of correct choices for Session 5, Mean ± SE: Control 50.00 ± 2.362; Trained 78.65 ± 2.992; N = 8, Supplementary Video S2). It should be noticed that all trained subjects accomplished the learning criterion and were included in further analysis. Here, we introduce the cue-guided rhomboid maze as a novel behavioral paradigm to assess spatial learning ability in adult zebrafish.

### Learning-Induced Cell Proliferation in Adult Zebrafish Pallium

Previous studies in goldfish reported that learning in the cue-guided rhomboid maze induces a selective increase of metabolic activity in the LP of trained subjects (Durán et al., 2015; Ocaña et al., 2017)⁠. Thus, we aimed to test whether learning in the rhomboid maze task would have any effect on cell proliferation in this neurogenic niche. It must be noticed that the Control group is subjected to manipulations, environment conditions and social reward in the same way as Trained subjects. Hence, changes in proliferation and/or neurogenesis should be related to the learning process.

To evaluate mitotic activity, we determined the expression of the Proliferating Cell Nuclear Antigen (PCNA) throughout the rostro-caudal axis of the pallium (Figures 1D–H, Supplementary Figures S1A–E). Expression of PCNA in the pallium was detected from the rostral to the caudal regions, in the periventricular zone. Supporting our hypothesis we found a learning-related increase in the PCNA detection in the LP, only restricted to its caudal region (cLP, ∼180%; Figures 1E,F). Unexpectedly, we also observed a relevant learning-related gain in PCNA levels in the rostral region of MP (rMP, ∼220%, Figures 1G,H).

It is known that senescence leads to cognitive deterioration, a fact that has also been proven for zebrafish (Ruhl et al., 2015; Adams and Kafaligonul, 2018; Yang et al., 2018)⁠. Thus, we explored whether aged zebrafish (21 months-old) could learn in the cue-guided rhomboid maze and, consequently, increase proliferation rates in the pallium. As observed for adult-young zebrafish, senescent individuals improve their task performance in a daily manner (Supplementary Figure S2). In agreement with our previous observations, aged fish exhibit an enhancement of PCNA expression exclusively in the rMP (∼180%) and the cLP (∼250%) of Trained fish (Supplementary Figure S2). Interestingly, we observed a slight improvement in the learning curve and in the basal levels of proliferation, in the aged zebrafish (Figure 1 and Supplementary Figure S2).

Our results indicate that cognitive activity, carried out on the cue-guided rhomboid maze, induces an increase in cell proliferation in two delimited neurogenic regions of the pallium of adult zebrafish.

### Enhanced Adult Neurogenesis by Learning in the Rhomboid Maze

**FIGURE 2:**
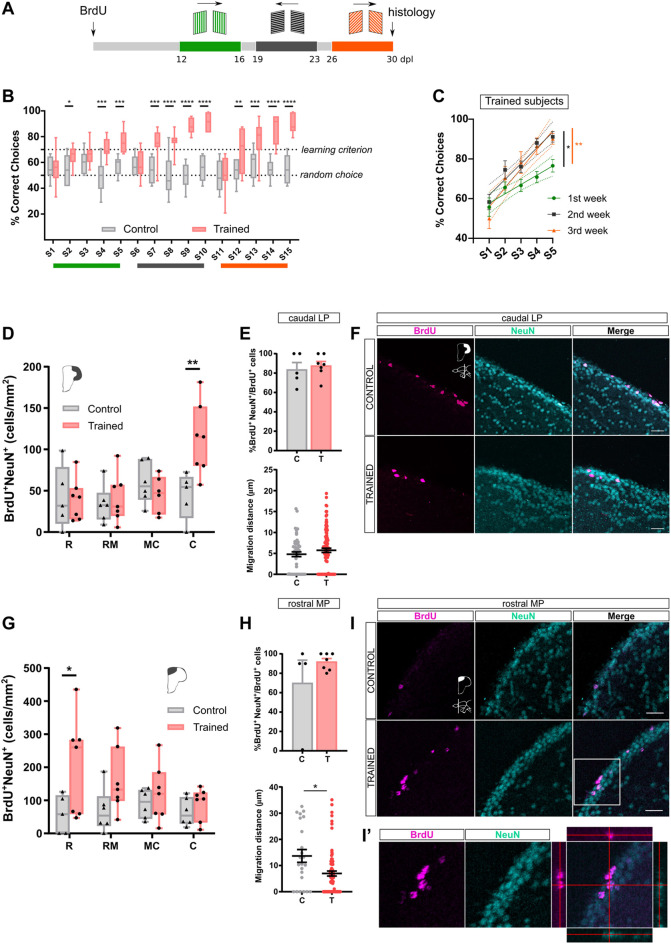
Sustained training of zebrafish from 12–30 dpl increases adult neurogenesis exclusively in the cLP and the rMP. **(A)**. Experimental schedule. Fish were immersed in BrdU, and training started at 12 dpl. Training consisted of 15 sessions, distributed in three slots. Every five sessions both cues and exit position were changed. A 2-day interval was fixed between slots. At 30 dpl fish were euthanised for histology. **(B)**. Learning curves for Trained and Control individuals (Two-way RM ANOVA, Treatment effect: F_(1, 14)_ = 67.71 with *p* < 0.0001, Session effect: F_(14, 196)_ = 6.805 with *p* < 0.0001. Bonferroni’s multiple comparisons test, * depicts *p* < 0.05, **p < 0.01, ****p < 0.0001. Trained, N = 8; Control N = 8). **(C)**. Simple linear regression for each training window for Trained fish (ANCOVA, first vs. 2nd week: F_(1,76)_ = 4.647; 1st vs. 3rd week: F_(1,76)_ = 7.068, * indicates *p* < 0.05, **, *p* < 0.01. Trained, N = 8; Control N = 8). **(D).** BrdU^+^NeuN^+^ cell quantification in LP (Two-way ANOVA, Treatment effect: F_(1, 41)_ = 2.766 with *p* = 0.103, Pallium region effect: F_(3, 41)_ = 4.493 with *p* = 0.0098. Bonferroni’s multiple comparisons test, **p < 0.01. Trained, N = 7; Control N = 6). **(E)**. Top: Neuronal fate (%BrdU^+^NeuN^+^/BrdU^+^ cells) in caudal LP (Unpaired *t* test, t_(10)_ = 0.5091, n. s. Trained, N = 7; Control N = 5). Bottom: Cell migration in caudal LP (Mann Whitney test, U = 2419, n. s. Trained, N = 7; Control N = 6). **(F)**. Adult-born neurons (BrdU/NeuN) in cLP for Trained and Control individuals. Scale bar, 20 μm. **(G).** BrdU^+^NeuN^+^ cell quantification in MP. (Two-way ANOVA, Treatment effect: F_(1, 43)_ = 9.564 with *p* = 0.0035, Pallium region effect: F_(3, 43)_ = 0.889 with *p* = 0.455. Bonferroni’s multiple comparisons test, *p < 0.05. Trained, N = 7; Control N = 6). **(H)**. Top: Neuronal fate (%BrdU^+^NeuN^+^/BrdU^+^ cells) in rMP (Mann-Whitney test, U = 11.5, n. s. Trained, N = 7; Control N = 4). Bottom: Cell migration in rMP (Mann Whitney test, U = 602, * depicts *p* < 0.05. Trained, N = 7; Control, N = 4). **(I).** Adult-born neurons (BrdU/NeuN) in rMP for Trained and Control individuals. Scale bar, 20 μm. **(I')**. Higher magnification of the boxed square in I (merge panel). Single focal plane and orthogonal views after three-dimension reconstruction.

Adult neurogenesis is a plastic phenomenon that can be modulated by network activity at different levels, such as NSCs proliferation, migration, differentiation and survival of new neurons. However, the role of cognitive activity on adult neurogenesis modulation has not been explored in zebrafish, yet. We hypothesized that sustained neuronal activity triggered as a consequence of learning impinges on immature adult-born neurons to promote pallial network remodeling. To approach this question we treated adult fish (∼10 months-old) with BrdU, a thymidine analogue, to label NSCs during the S-phase of mitotic cell division. We allowed BrdU-labeled neurons to develop over a 12-day period. Then, fish were randomly divided into Trained and Control (mocked) groups, and subjected to sustained training in the rhomboid maze from 12 to 30 dpl, a period in which we estimate that adult-born neurons are maturing (Figure 2A). To maintain the cognitive challenge during the training period, we changed the cues and the learning rule at the beginning of each week. Trained subjects exhibited a good learning curve, reaching the established criterion at the fifth session of the first week, and were able to relearn the task after the rule shift during the following weeks (Figure 2B, Percentage of correct choices for Session 5, Mean ± SE: Control 57.81 ± 2.54; Trained 76.56 ± 3.14, N = 8). Notably, after the first week of training, the subjects showed a better performance when relearning the task, evidenced by an increase in the learning curve slope of Trained fish (Figure 2C, Simple linear regression, 1^st^ week vs. 2 weeks: *p* = 0.0343; 1^st^ week vs. 3^rd^ week: *p* = 0.0096).

After completing the training schedule, we assessed the fate of BrdU-labeled cells by immunodetection of the neuronal marker NeuN. The effect of learning on adult neurogenesis was evaluated by quantifying the number of adult-born neurons (BrdU^+^NeuN^+^) throughout the pallial rostro-caudal axis. We found that the cLP of Trained subjects exhibited an increase (∼ 250%) in the number of 30 dpl BrdU-labeled neurons as compared to Control group, whereas the other rostro-caudal LP regions shown similar levels of 30 dpl adult-born neurons between both experimental groups (Figures 2D,F, BrdU^+^NeuN^+^ in cLP, Mean ± SE: Control 44.39 ± 12.71, N = 5; Trained 112.28 ± 16.41, N = 7). Similarly, in the MP we observed an increase of BrdU-labeled neurons (> 300%) exclusively in the rostral region of the MP (Figures 2G,I,I’, BrdU^+^NeuN^+^ in rMP, Mean ± SE: Control 57.73 ± 26.00, N = 5; Trained 204.50 ± 56.36, N = 7). Since network activity may regulate adult-born neurons development, we decided to evaluate if learning affects neuronal differentiation, assessed as the percentage of labeled cells which adopt neuronal phenotype (BrdU-labeled cells that express the neuronal marker NeuN, BrdU^+^NeuN^+^/BrdU^+^), as well as the migration distance of BrdU-labeled cells, measured from the ventricular boundary into the final position in the pallial parenchyma (Supplementary Figure S3). We analyzed only the regions in which learning promotes adult neurogenesis. Neither of these parameters were affected by learning, except for the migration distance in rMP, which was lower in Trained subjects (Figures 2E,H). The difference in migration distance may account for late recruited BrdU-labeled progenitors, which were product of conservative division and now are being activated by learning, a concept that we are exploring below. Thus, it is expected that younger BrdU-labeled neurons remains close to the periventricular zone. These results indicate that training in the rhomboidal maze task during a 12–30 dpl time frame promotes adult neurogenesis in the cLP and in the rMP, shedding light on the potential relevance of these pallial regions during learning.

### Critical Period for Learning-Induced Adult Neurogenesis

**FIGURE 3:**
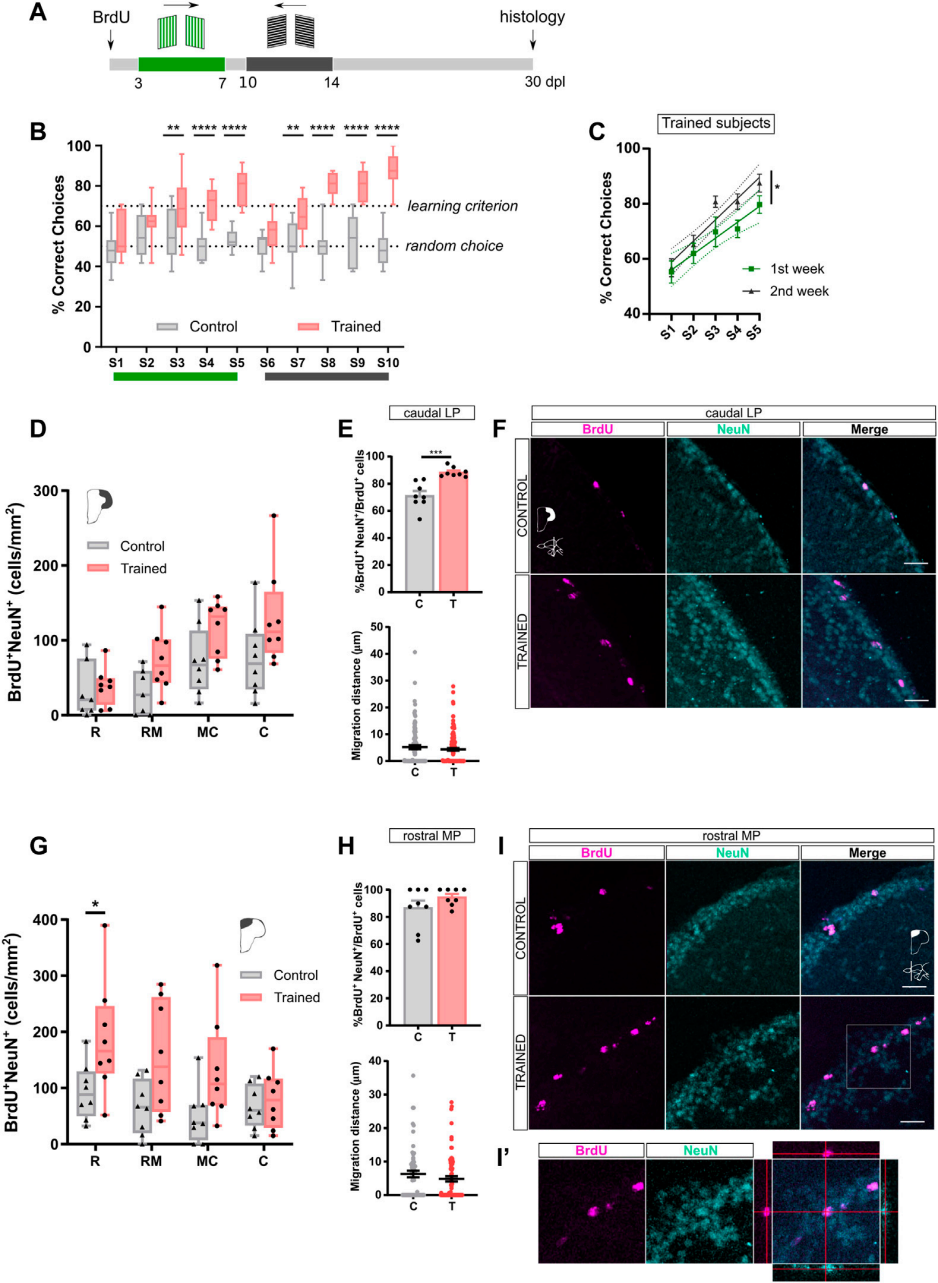
Training zebrafish during an early period (3–14 dpl) increases adult neurogenesis in the rMP. **(A)**. Experimental design. **(B)**. Learning curves for Trained and Control individuals (Two-way RM ANOVA, Treatment effect: F_(1, 14)_ = 126.1 with *p* < 0.0001, Session effect: F_(9, 126)_ = 5.254 with *p* < 0.0001. Bonferroni’s multiple comparisons test, *** depicts *p* < 0.001, ****, *p* < 0.0001. Trained, N = 8; Control N = 8). **(C)**. Simple linear regression for each training window for Trained fish (ANCOVA, F_(1, 76)_ = 1.516, n. s. Trained, N = 8; Control N = 8). **(D)**. BrdU^+^NeuN^+^ cell quantification in LP. (Two-way ANOVA, Treatment effect: F_(1, 54)_ = 10.63 with *p* = 0.0019, Pallium region effect: F_(3, 54)_ = 8.642 with *p* < 0.0001. Bonferroni’s multiple comparisons test, not significant differences. Trained, N = 8; Control N = 8). **(E)**. Top: Neuronal fate (%BrdU^+^NeuN^+^/BrdU^+^ cells) in cLP. (Unpaired *t* test, t_(14)_ = 4.749, *** depicts *p* < 0.001. Trained, N = 8; Control N = 8). Bottom: Cell migration in cLP (Mann Whitney test, U = 6492, n. s. Trained, N = 8; Control N = 8). **(F)**. Adult-born neurons (BrdU^+^NeuN^+^) in cLP for Trained and Control individuals. Scale bar, 20 μm. **(G)**. BrdU^+^NeuN^+^ cell quantification in MP. (Two-way ANOVA, Treatment effect: F_(1, 56)_ = 15.34 with *p* = 0.0002, Pallium region effect: F_(3, 56)_ = 2.685 with *p* = 0.0553. Bonferroni’s multiple comparisons test, * depicts *p* < 0.05. Trained, N = 8; Control N = 8). **(H)**. Top: Neuronal fate (%BrdU^+^NeuN^+^/BrdU^+^ cells) in rMP (Unpaired *t* test, t_(14)_ = 1.396, n. s. Trained, N = 8; Control N = 8). Bottom: Cell migration in rMP (Mann Whitney test, U = 2088, n. s. Trained, N = 8; Control N = 8). **(I)**. Adult-born neurons (BrdU^+^NeuN^+^) in rMP for Trained and Control individuals. Scale bar, 20 μm. **(I')**. Higher magnification of the boxed square in I (merge panel). Single focal plane and orthogonal views after three-dimension reconstruction.

Our previous results indicate that learning has an immediate effect on cell proliferation in the cLP and rMP neurogenic regions (Figure 1). Also we found a learning-related increase of adult-born neurons in the in the same pallial regions after a 12–30 dpl training (Figure 2). The neurogenic process involves several checkpoints where network activity and systemic signaling regulate distinct developmental processes in a time-dependent manner (Mu et al., 2010)⁠. On these basis, we evaluated if learning has an impact on adult-born neurons at different maturation periods. We did not observe significant changes between the Control and Trained groups at any rostro-caudal region of LP when training occurred during an earlier maturation stage of adult-born neurons (3–14 dpl, Figures 3A–D,F). On the other hand, we found that learning stimulates an increase (∼200%) in the levels of rMP 30 dpl adult-born neurons (Figures 3G,I,I’, BrdU^+^NeuN^+^ in rMP, Mean ± SE: Control 94.20 ± 17.61; Trained 188.30 ± 36.01, N = 8). Furthermore, we evaluated neuronal fate and observed a significant increase in the percentage of newborn neurons only in the cLP. The final position of the labeled neurons (migration distance) showed no differences at any region (Figures 3E,H).

**FIGURE 4:**
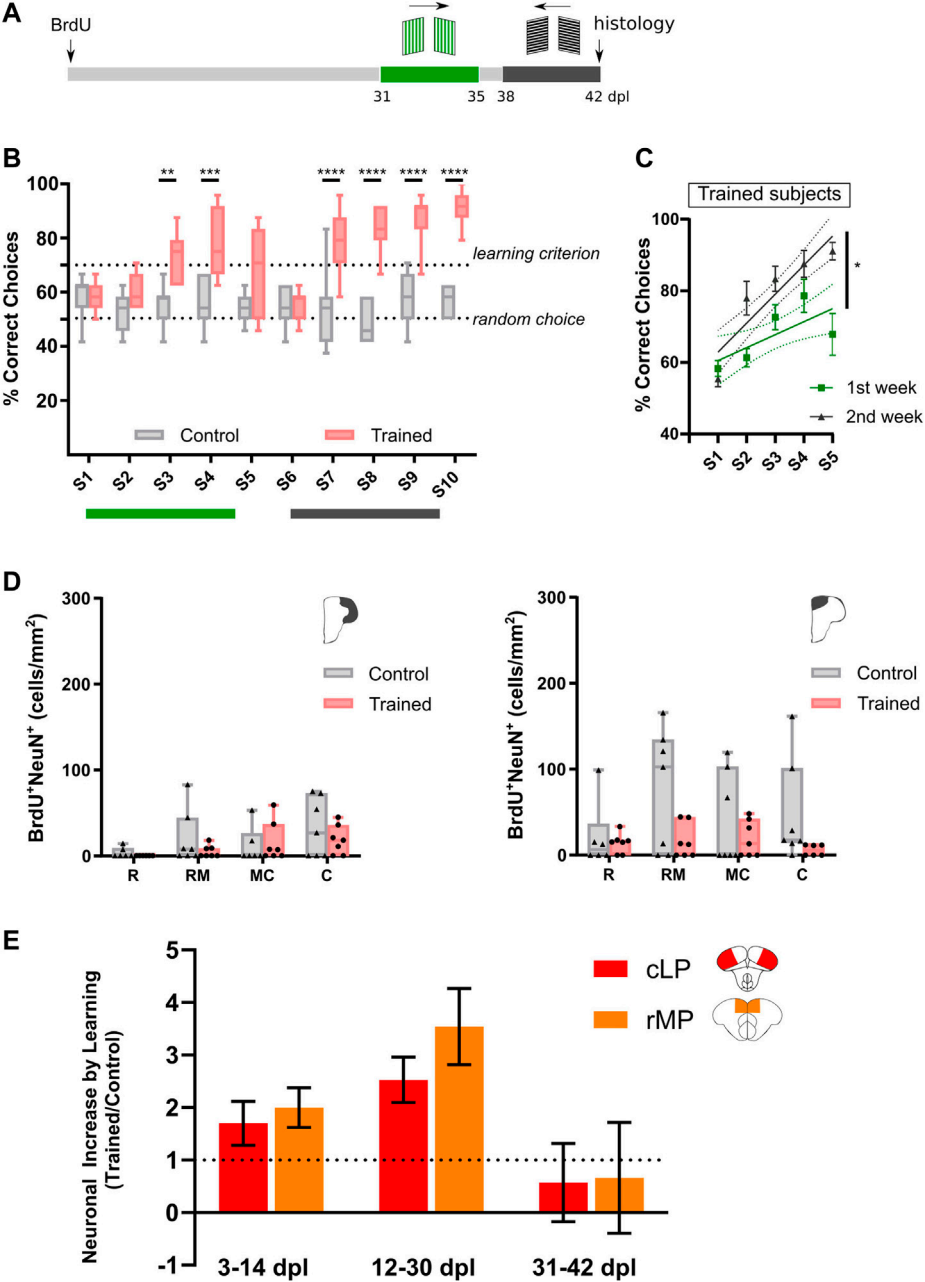
Training adult zebrafish during a late period (31–42 dpl) has no effect on pallial neurogenesis. **(A)**. Experimental design. **(B)**. Learning curves for Trained and Control individuals. (Two-way RM ANOVA, Treatment effect: F_(1, 12)_ = 108.0 with *p* < 0.0001, Session effect: F_(9, 108)_ = 6.800 with *p* < 0.0001. Bonferroni’s multiple comparisons test, **p < 0.01, ***p < 0.001, ****p < 0.0001. Trained, N = 7; Control N = 7). **(C)**. Simple linear regression for each training window for Trained fish (ANCOVA, F_(1, 66)_ = 5.914, * indicates *p* < 0.05. Trained, N = 7; Control N = 7). **(D)**. BrdU^+^NeuN^+^ cell quantification in LP (left) and MP (right) (Kruskal–Wallis test with: Left panel, *p* = 0.1984; Right panel, *p* = 0.316. Dunn’s multiple comparisons test, not significant differences were found. Trained, N = 7; Control, N = 7). **(E)**. Neuronal increase ratio (Trained/Control) in rMP and cLP for each learning time frame.

Next, we assessed adult neurogenesis after training fish during a late period, at 31–42 dpl (Figures 4A–C). Both experimental groups exhibited similar levels of neurogenesis (Figure 4D). Our results indicate that adult neurogenesis in the rMP and the cLP is sensitive to training in the rhomboid maze, during restricted periods (Figure 4E). The critical period for learning-induced adult neurogenesis is slightly shorter in the cLP as compared to the rMP. This modulation of adult neurogenesis by network activity could be attributed to two possible non-excluding mechanisms: 1) activity-related increase in the survival of adult-born neurons, avoiding cell death programs; 2) activity-related chained recruitment of BrdU-labeled cells, which were product of conservative divisions.

### Chained Proliferation of Labeled NSCs and Adult-Born Cell Death Modulate Adult Neurogenesis in the Pallium

**FIGURE 5:**
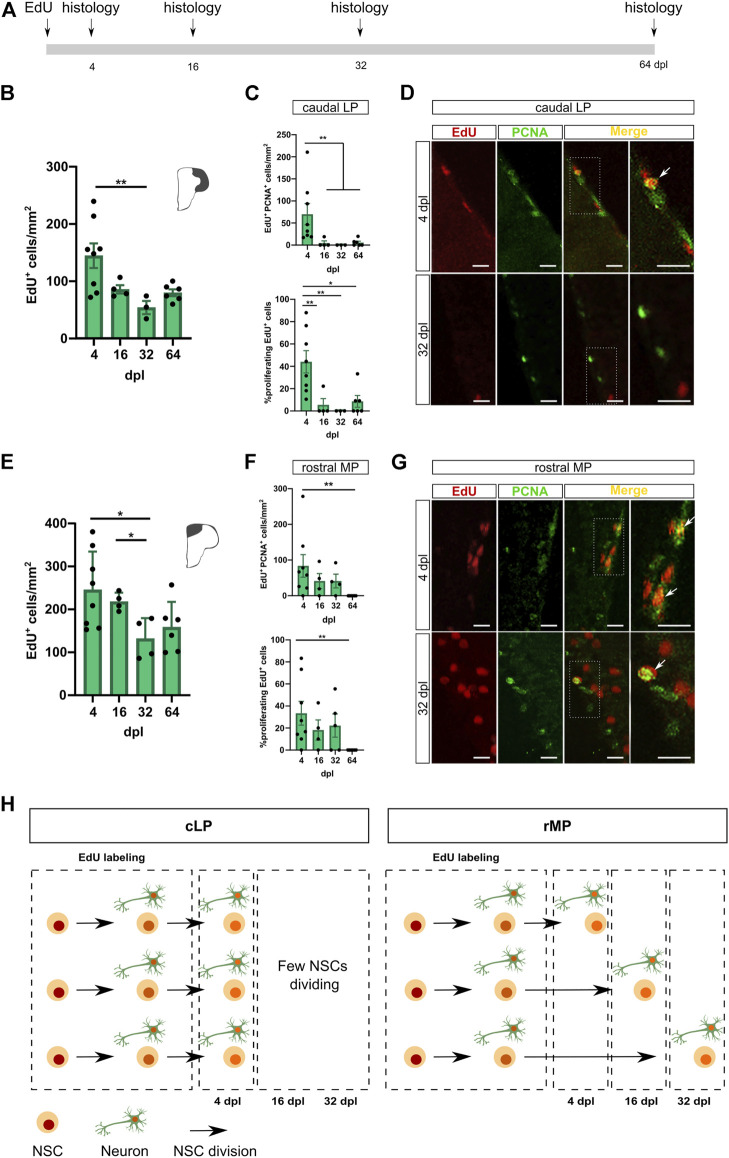
Balance of adult neurogenesis driven by chained proliferation and loss of adult-born cells. **(A)**. Experimental design. Fish were i. p. injected with EdU and euthanised for histology at three times points: 4, 16, 32 and 64 dpl. **(B)**. EdU^+^ cell quantification in cLP in 4, 16, 32 and 64 dpl. (Kruskal–Wallis test, followed by post-hoc Dunn’s test, K-W st = 9.048, ** depicts *p* < 0.01. 4 dpl, N = 8; 16 dpl, N = 4; 32 dpl, N = 3, 64 dpl, N = 6). **(C)**. Number of proliferating EdU cells (top) and % of proliferating EdU cells (bottom) (Kruskal–Wallis test, followed by post-hoc Dunn’s test, K-W st = 13.80 and K-W st = 12.88, respectively. * depicts *p* < 0.05, **, *p* < 0.01. 4 dpl, N = 8; 16 dpl, N = 4; 32 dpl, N = 3, 64 dpl, N = 6). **(D)**. EdU/PCNA in cLP for 4 dpl and 32 dpl. Scale bar, 20 μm. **(E)**. EdU^+^ cell quantification in rMP in 4, 16, 32 and 64 dpl. (Kruskal–Wallis test, followed by post-hoc Dunn’s test, K-W st = 7.88, * depicts *p* < 0.05. 4 dpl, N = 8; 16 dpl, N = 4; 32 dpl, N = 4, 64 dpl, N = 6). **(F)**. Number of proliferating EdU cells (top) and % of proliferating EdU cells (bottom) (Kruskal–Wallis test, followed by post-hoc Dunn’s test, K-W st = 9.571 and K-W st = 8.827, respectively. ** depicts *p* < 0.01. 4 dpl, N = 8; 16 dpl, N = 4; 32 dpl, N = 3, 64 dpl, N = 6). **(G)**. EdU/PCNA in rMP for 4 dpl and 32 dpl. Scale bar, 20 μm. **(H)**. Scheme summarizing EdU/PCNA results in cLP and rMP.

In rodents, it is well established that network activity promotes synaptic integration of adult-born neurons and, in consequence, favor the neuronal survival (Ryu et al., 2016)⁠. Hitherto, this hypothesis has not been explored in teleost fish, yet. On the other hand, Than-Trong and coworkers (2020) proposed that adult neurogenesis relies on different kinds of NSCs, a reservoir pool involved in the self-renewal of NSCs (rNSC), and an operational group with neurogenic function (oNSC) (Than-Trong et al., 2020)⁠. The oNSCs can divide either in a symmetric or asymmetric way to produce new neurons and to preserve the NSC pool. In this context, our adult neurogenesis results would rely on learning-induced sequential recruitment of BrdU-labeled cells or, alternatively, on an activity-dependent rescue from death. Therefore, we assessed a time course for neuronal survival and proliferation of EdU-labeled cells at four temporal points: 4, 16, 32 and 64 dpl (Figure 5A).

We found a ∼37% neuronal survival in the cLP when comparing 4 vs. 32 dpl (Figures 5B,D, Number of EdU^+^ cells in cLP: 4 dpl 144.7 ± 21.53, N = 8; 32 dpl 54.16 ± 11.47, N = 3), whereas for rMP the survival of EdU-labeled cells during the same period was ∼55% (Figures 5E,G, Number of EdU^+^ cells in rMP: 4 dpl 245.6 ± 31.45, N = 8; 32 dpl 131.8 ± 23.84, N = 4). The decrease in the number of labeled cells in both pallial regions could indicate the death of a portion of these cells. Furthermore, we assessed the sequential recruitment of EdU-labeled cells by PCNA expression. We found a relevant fraction (44.03 ± 10,02%) of mitotic EdU-labeled cells at 4 dpl in the cLP (Figure 5C), which diminished considerably at later times. In contrast, in the rMP we found a portion of EdU^+^ proliferating cells from 4 to 32 dpl (26.54 ± 6.21%), while we observed scarce proliferation in labeled cells at 64 dpl. (Figure 5F). Hence, a considerable portion of proliferating cells retain EdU labeling and continues dividing in the cLP and the rMP (Figure 5H). Our results point to a complex regulation of adult neurogenesis in the pallium of zebrafish, where chained proliferation of NSCs and death of immature neurons are balanced to contribute to the neuronal addition on these networks.

### A Population Dynamics Model Mimics the Learning-Induced Adult Neurogenesis in the MP

**FIGURE 6:**
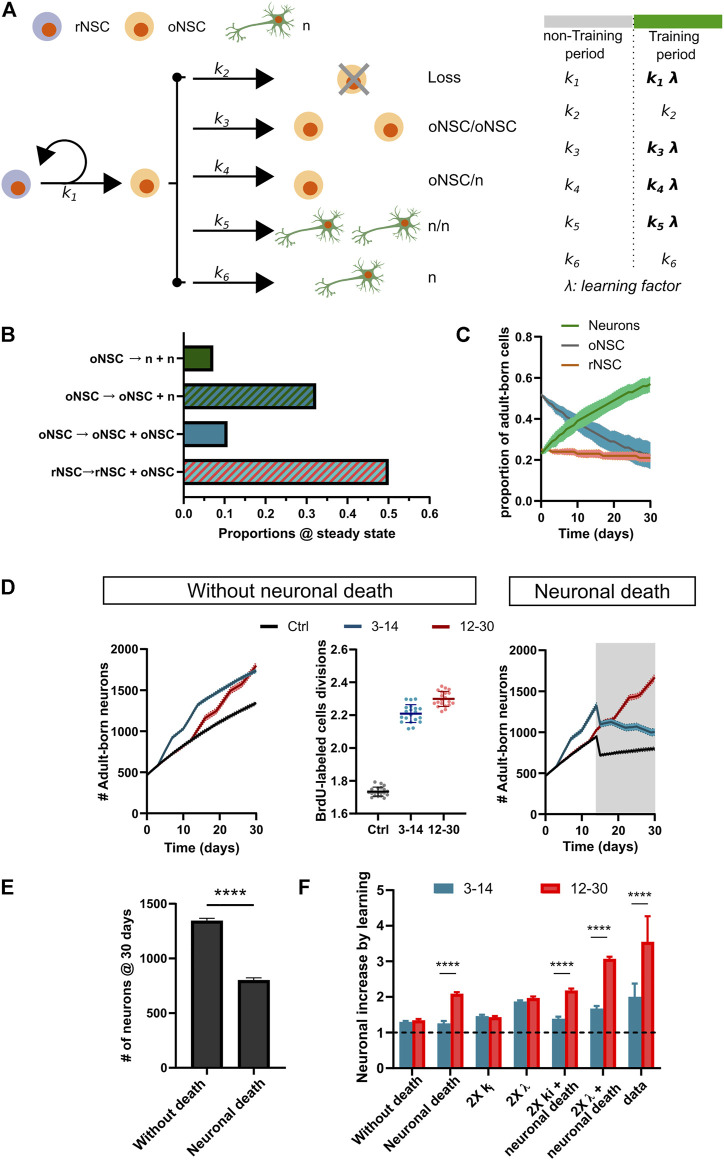
A population NSC dynamics model mimics the learning-induced adult neurogenesis in the rMP. **(A)**. Scheme outlining NSC population dynamics model. ki indicates rates of division/differentiation. The learning effect on NSC proliferation was simulated by adding a learning factor (*λ*) during training windows. rNSC: reservoir neural stem cell; oNSC: operative neural stem cell; n: neuron. **(B)**. Number of cellular divisions at steady state, calculated to estimate the initial populations of BrdU-labeled cells. **(C)**. Population dynamics under Control condition. **(D)**. Left: Stochastic simulations to estimate the population of adult-born neurons in Control and Trained subjects during two training periods (3–14 dpl) and three training periods (12–30 dpl). Middle: BrdU-labeled cells division under Control and Training conditions. Right: Stochastic simulations to estimate the population of adult-born neurons considering a checkpoint (at 15 dpl) for neuronal death, together with a learning-induced rescue. **(E)**. Adult-born neurons for Control subjects at 30 dpl, with and without neuronal death (Unpaired *t*-test, t_(38)_ = 73.10, **** depicts *p* < 0.0001, N = 20). **(F)**. Neuronal increase by learning under different conditions (Two-way ANOVA, Period effect: F_(6, 239)_ = 430.7 with *p* < 0.0001, Condition effect: F_(1, 239)_ = 1,342 with *p* < 0.0001. Post-hoc Sidak’s test **** depicts *p* < 0.0001- N = 20 for simulation, N = 5 for data).

Recently, Than-Trong and coworkers (2020) performed an *intra-vital* imaging analysis to track the fate of NSCs in the MP. Based on the experimental results and a computational model, the authors estimated the proportions of symmetric and asymmetric divisions, and possible fates adopted by activated NSCs. Based on the division rates observed by Than-Trong and coworkers, we adapted their model to assess the dynamics of neuronal addition under different conditions: Control and Trained at different learning periods (Figures 6A,B). After 30 days, the model evolves to a proportion ∼60% of adult-born neurons from the initial cohort of “labeled” NSCs in the rMP (Figure 6C), a value slightly lower to what is shown in our experiments (Panels E, H from Figures 2, 3). Next, we calculated the number of chained divisions that each cell of the original NSC pool goes through. The model indicates an average of ∼1.7 divisions during the 30-day period (Figure 6D), a value that supports the chained proliferation of labeled-NSCs by discarding a relevant BrdU dilution in the progeny. Then, based on our PCNA results (see Figure 1), we hypothesized that learning would burst the activation and proliferation of BrdU-labeled NSCs, which were a product of NSC-conservative divisions from the original labeled pool. We aimed to emulate the neuronal population dynamics in rMP when learning occurs from 3–14 dpl (2 weeks of training) and from 12–30 dpl (3 weeks of training), the experimental conditions in which learning promotes adult neurogenesis. Thus, during these training windows, the proliferation rate of NSCs is affected by a learning factor (*λ*). We observed that learning increased the number of adult-born neurons at the expense of the operative NSC pool (Figure 6C). However, in contrast to the changes observed in our experiments (See Figure 4E), in the simulation both training conditions (3–14 and 12–30 dpl) exhibited a similar outcome in the number of new neurons (Figure 6D). Since we observed neuronal loss in the rMP from 4 to 32 dpl (indicated by ∼45% reduction in the number of EdU-labeled cells), we incorporated to our model a checkpoint starting at 15 dpl to allow the survival or death of new neurons, together with a learning-related rescue (See methods). In Control conditions the death of adult-born neurons maintains a 67.1 ± 5.4% of survival (Figure 6D,E). As expected, the incorporation of neuronal death to the model increases the difference in the number of adult-born neurons when training occurs during 12–30 dpl in comparison with 3–14 dpl (Figure 6D). This learning-induced adult neurogenesis profile mimics our experimental results, but with a lower difference than our experimental data. This result, led us to interrogate the model under different conditions, as duplication of the learning factor (2X *λ*), duplication of all the division/differentiation rates (2X k_i_), 2X *λ* + neuronal death, and 2X k_i_ + neuronal death (Figure 6F, Supplementary Figure S5A). The only conditions where the model mimicked the experimental data profiles were the ones in which neuronal death was taken into account, being the 2X *λ* + neuronal death the most accurate condition. A long-term simulation (500 days) showed that adult neurogenesis is additive to pallial networks, even when considering neuronal death (Supplementary Figure S5B). Thus, the model supports that learning-induced adult neurogenesis in rMP relies on both mechanisms: a boost in chained NSC proliferation and rescue from neuronal loss.

## Discussion

Teleost fish grow throughout their lives, therefore their organs must adapt to their increasing body size (Jerison, 1973)⁠. Consequently, adult neurogenesis could be considered as a mechanism that underlies the constant growth of the fish brain, in agreement with the numerical matching hypothesis (Zupanc, 2021)⁠. As an alternative but not excluding hypothesis, adult neurogenesis would provide neural networks with an extra degree of plasticity to adapt the brain to changes in the environment. Previous studies in teleosts suggest functional specialization of neurogenic niches (Zupanc and Horschke, 1995; Kaslin et al., 2008; Iribarne and Castelló, 2014; Lindsey et al., 2014; Olivera-Pasilio et al., 2014; Sato et al., 2017; Labusch et al., 2020)⁠. These studies demonstrate that different brain regions involved in the processing of sensory activity are neurogenic, and sustained sensory stimulation leads to an increase in newborn neurons only in the related niches. In the same way, in rodents and birds it has been shown that behavioral challenges involving information processing in neurogenic brain nuclei enhance adult neurogenesis in a stimulus-dependent fashion (Goldman and Nottebohm, 1983; Barnea et al., 1994; Leuner et al., 2004; Alonso et al., 2006; Tashiro et al., 2007)⁠. These works highlight the relevance of adult neurogenesis on learning-related changes in a structure-to-function manner. Here, we challenged zebrafish with a cognitive paradigm to explore the addition of adult-born neurons to pallial circuits. The subjects were trained in a spatial learning paradigm to integrate their positional information with visual cues to solve the task. To avoid egocentric responses, the start compartments were randomly chosen and multiple maze rotations were performed during sessions. Therefore, on each trial, the fish must make a decision based on spatial information. Adult zebrafish exhibited good performance on this behavioral test, even after sequential rule-change sessions. We observed that training fish in this paradigm increases cell proliferation in circumscribed pallial subregions. The Control group, mocked with a random exit, was subjected to all the experimental manipulations (isolation, handling, environment, exploration, and social reward) as the Trained group. Hence, we conclude that the observed differences in adult neurogenesis are a consequence of the learning process. The Trained subjects evidenced a region-specific increase in the rate of proliferation when compared to Control fish. This finding suggests that the new neurons could underlie plastic changes in the pallium as a consequence of the cognitive challenge.

Our results indicate a rostro-caudal specialization in the pallial circuits, shedding light on the relevance of the cLP (encompassing the caudal Dlv and Dld) and the rMP (the rostral portion of Dm) during the execution of the cognitive task implemented here. Behavioral studies involving distinct teleost fish related the LP to navigation and spatial learning (Durán et al., 2015; Elliott et al., 2017; Ocaña et al., 2017; Fotowat et al., 2019)⁠. Both of these cognitive functions are processed by the mammalian hippocampus. Furthermore, the zebrafish LP region expresses several molecular markers resembling the ones expressed by the mammalian hippocampus (Mueller and Wullimann, 2009; Ganz et al., 2015)⁠. Our results are in agreement with the involvement of the teleost LP in processing spatial information and reveal a rostro-caudal specialization of this structure, being the cLP the only region in which this spatial task heightens the addition of new neurons. In this regard, Ocaña and coworkers (2017) reported progressive changes in metabolic activity throughout the rostro-caudal LP as a response to training goldfish in the same paradigm. In their work, oxidative metabolism activity was analyzed during learning showing that rostral and medial sections of Dlv exhibit transient activation at early stages, whereas the caudal Dlv shows sustained activity throughout the training period. In our experiments we found no differences between the dorsal and ventral LP (Dld and Dlv, data not shown) and only observed learning-related effects on adult neurogenesis in the cLP. Very likely, the signaling involved in oxidative metabolism during this paradigm could be different than the factors involved during neurogenesis in the LP, explaining the discrepancies. Unexpectedly, we also found a learning-related induction of adult neurogenesis in the rMP, a brain region that has been poorly studied in teleosts. Interestingly, Lau and coworkers (2011) have proposed that Dm acts as a brain center whose activity discriminates a choice behavior in zebrafish (Lau et al., 2011)⁠. In their work the authors analyzed neuronal activity by c-fos expression in the pallium, but only at a rostral level (rMP, slice 71 of the zebrafish atlas (Wullimann et al., 1996)⁠). On this basis, we hypothesize that the rMP learning-related increase in adult neurogenesis could be attributed to the role of this neural center on decision-making. However, future experiments should be conducted to test this idea.

The pallium of fish is considered a simple structure with specialized regions at the transversal level. However, here we observed a rostro-caudal specialization in the MP and the LP of Control fish, evidenced by gradients of PCNA (Figure 1), with higher proliferation activity in the rMP and the cLP, being both regions prone to be modulated by learning. Interestingly, our results highlight the specialization of neural circuits along the pallial rostro-caudal axis, a concept that should be taken into account in future studies.

To evaluate the learning effects on adult neurogenesis, we labeled proliferating progenitor cells with the thymidine analog BrdU and fish were trained at different periods after progenitor labeling (3–14, 12–30, 31–42 dpl). After a single thymidine analog pulse (BrdU or EdU), most of the label will be incorporated by the active NSC (5% of total NSCs, (März et al., 2010; Than-Trong et al., 2020),⁠) and by a fraction of neuronal progenitors (∼6–26% (März et al., 2010; Rothenaigner et al., 2011)⁠). After short chase times, most adult-born neurons will be the product of neurogenic divisions by the committed neuronal progenitors. While, the late recruitment of adult-born neurons (here described as chained proliferation) would result from conservative NSC divisions at the time of BrdU administration, which will go through consecutive mitosis and differentiation beyond neurogenic fate.

We found an increase of BrdU-labeled neurons in the rMP and cLP of Trained subjects. Our results support adult neurogenesis as an evolutionary conserved source of learning-related brain plasticity. Interestingly, the neurogenesis observed in these pallial regions differs in their learning-sensitive critical periods, where the cLP has a shorter temporal window as compared to the rMP. The distinct critical periods between these pallial regions could be attributed to different composition of NSC and progenitor cells between both niches. Or alternative, could be explained by neuronal populations with a distinct maturation pace. In rodents, several activity-dependent critical periods have been reported during development of adult-born neurons (Ge et al., 2007; Tashiro et al., 2007; Alvarez et al., 2016)⁠; however to our knowledge this is the first evidence reported in a teleost model.

The observed increase in adult neurogenesis after the cognitive challenge could underlie two distinct, yet not exclusive, mechanisms: 1) rescue of immature neurons from death programs; or 2) an expansion of the labeled NSC reservoir by chained recruitment of BrdU-labeled cells. Here, we found that both processes could contribute to adult neurogenesis homeostasis in the zebrafish pallium (Figure 6). In rodents, adult neurogenesis generates neurons in abundance, and their survival depends on an activity-dependent synaptic integration of new neurons to rescue them from death programs (Ryu et al., 2016)⁠. In line with this idea, we found a decrease in the number of adult-born neurons in cLP and rMP over a 64-days lapse (Figures 5B,E). Accordingly, Ampatzis and coworkers (2012) showed significant apoptotic activity assessed by TUNEL method in different regions of adult zebrafish pallium (∼100–200 TUNEL profiles/mm^2^, a value close to the daily adult neurogenesis contribution) (Ampatzis et al., 2012)⁠. On the other hand, supporting the chained proliferation, we found significant amounts of EdU^+^PCNA^+^ cells in both pallial subregions (Figures 5C,F). Whereas the rMP maintains a steady level of cell proliferation over the 4–32 days period, the cLP shows an early boost of proliferation activity, which declined by day 16. The differences in the proliferation dynamics between both sub-regions could be attributed to heterogeneous cellular compositions (März et al., 2010; Lindsey et al., 2012; Dirian et al., 2014; Anand and Mondal, 2017)⁠. Thus, at 4 dpl double-labeled cells in rMP and cLP may correspond to fast-cycling cells (potentially intermediate amplifier progenitors or transitory amplifying cells), which proliferate over a short period. In cLP, there is scarce proliferation activity detected after 4 dpl. However, in the rMP, a relevant portion of EdU^+^ cells continues proliferating at 16 and 32 dpl. These may correspond to slow-cycling cells, which re-entered the cell cycle after a quiescent state (Alunni et al., 2010; Olivera-Pasilio et al., 2014)⁠. The fast decrease, after 4 dpl (Figure 5C), in chained proliferation observed in cLP could explain the absence of learning effects when subjects were trained at 3–14 dpl. While the late boost (12–30 dpl) of adult neurogenesis in this region (Figure 2D), could be attributed to an activity-dependent rescue from cell death programs. On the other hand, in rMP both chained proliferation and death rescue seem to be synergistic to the learning-related increase of 12–30 dpl adult-born neurons.

In agreement with our observations and supporting a chained proliferation of pallial NSCs, a persisting proliferation of BrdU-labeled cells (∼40 dpl) in the zebrafish telencephalon was also reported by other authors (Grandel et al., 2006; März et al., 2010)⁠. The chained proliferation of labeled NSCs has also been proposed by Prickaerts and collaborators (Prickaerts et al., 2004)⁠. In their words, “the effect of proliferation alone, on every day after injection, is added to the number of cells counted as well as the survival of those cells which were labeled earlier in the week and have not continued to proliferate”. Therefore, our results argue in favor of complex regulation of adult neurogenesis in which learning promotes the chained division of NSCs, together with activity-dependent survival. However, it is not clear whether new-born neuron integration and survival is related to NSC proliferation in a causative manner, a question that should be addressed in future research. The concept of chained-proliferation is supported by our NSC population dynamics model, in which proliferation and death act in a synergistic way to induce pallial circuit modifications by adult neurogenesis. Although the model reproduces the adult neurogenesis profiles observed in our experimental conditions, the proportion of adult-born neurons as well as its learning-induced increase exhibit lower values when compared to our experimental data. We speculate that these discrepancy could be related to different factors, such as different division/differentiation (k_i_) rates in our fish as compared to the ones calculated in the Than-Trong work, underestimation of the learning factor (*λ*), or the impact of neuronal death implemented in our model.

Taken together our results indicate that learning in a cognitive paradigm, involving spatial and positional information together with decision making, induces the addition of new neurons into specific pallial circuits. However, from our experiments, it is not clear whether neurons generated during training would participate in encoding information related to the learning process itself, since these new neurons could still be in an immature stage. If this is true, then the learning-induced neurogenesis would prepare the related neuronal circuits by adding new neurons for future challenges. In line with this idea, it was recently shown that silencing hippocampal adult-born neurons, which were immature during learning, impairs remote memory reconsolidation in rats, indicating a learning-related priming of immature neurons (Lods et al., 2021)⁠. This idea, as well as elucidating the timing for maturation and synaptic integration of adult-born neurons should be explored in future works.

## Conclusion

The addition of adult-born neurons by adult neurogenesis represents a major source of brain plasticity. Although zebrafish possess high levels of adult neurogenesis broadly distributed throughout their brain, the involvement of neuronal addition as a cognitive-related plastic mechanism has not been explored in this model, yet. The zebrafish pallium has numerous well described neurogenic niches, and has been proved to be critical for the execution of spatial and emotional learning tasks. In this work, we trained adult zebrafish in a cue-guided maze and found an improvement in their performance in a daily manner throughout five sessions. This cognitive challenge induces an increase in proliferation activity only in two restricted pallial areas, the cLP and the rMP. In addition, adult-born neurons in rMP and cLP are being produced on demand during the learning process but with distinct critical periods. Finally, based on a NSC population dynamics model we propose that adult neurogenesis is regulated in a complex manner by promoting NSC proliferation together with neuronal death programs. We propose that both processes are prone to be regulated by learning-induced network activity.

## Methods

**Subjects and housing.** All experiments were carried out using 10 ± 1 months-old AB-wild type zebrafish (*Danio rerio*) line in AB background, except for the experiment shown in Supplementary Figure S2, in which 21 months-old individuals were used. Adult zebrafish were housed in a zebrafish standalone rack system (ZS560, Aquaneering Inc.), were fish were kept in small groups (5 fish/l) with aerated and filtered water at a constant temperature of 27 ± 0.5°C, pH = 7.2 ± 0.2. The aquarium room was subjected to a 14:10 h light/dark cycle. Dry food and *Artemia salina* were provided three-times a day. During the experimental period fish were housed individually in 1.4 L tanks. Both sexes were used indistinguishably. Experimental procedures were conducted in accordance with the National regulations and following the Universities Federation for Animal Welfare Handbook on the Care and Management of Laboratory and other Research Animals. This work has the consent of the Comisión Nacional de Energía Atómica’s IACUC, protocol #05-2018-02.

**Behavioral paradigm.** Subjects were trained in a square tank (30 × 30 cm) containing the experimental rhomboid maze in the center, as was previously described (Ingle and Sahagian, 1973)⁠. The maze was made out of green PVC (10 × 10 cm), to form a diamond-shaped box with two circular starting compartments in opposite corners. The remaining corners of the box served as exits. One of them was kept open, while the other was blocked with a transparent glass barrier. Spatial cues, consisting of removable striped panels, were placed on two walls of the box. On the edges of the tank, and behind the starting compartments, two glass enclosures containing conspecifics (two per enclosure) were used as social reward. The experimental tank was illuminated with two LED lamps, located above the tank.

Two days prior to training, subjects were habituated to the experimental apparatus. During habituation fish were allowed to swim through both exits of the apparatus. Neither the cues nor the glass barrier were used during habituation. For individual habituation sessions conspecifics were also placed in the tank enclosures. After habituation, the training sessions were carried out. Each training session consisted of an acclimation period (5 min) followed by a maze solving period (5 min), which included chasing and capturing the fish with a small plastic vessel. Each session consisted of 24-trial trials, and was divided into two slots, one in the morning and the other in the afternoon to avoid exhaustion. For each trial, fish were randomly placed in either of the starting compartments (50% each one), and allowed to enter the central arena by removing a sliding PVC barrier. The initial decision of the fish was registered. A correct choice was scored when it swam through the exit, and a failure if it bumped against the glass barrier. In case of a failure, the experimenter waited until the fish found the correct exit. Once out of the maze, the fish were allowed to explore the tank and conspecifics for 10 s. Learning criterion was established at 70% of correct choices. The maze, cues and glass barrier were consistently rotated every five trials to avoid the use of external cues. In the Control group, the glass barrier was randomly placed in any of the exits. Other from that, conditions remained the same as the Trained group.

**BrdU labeling.** To label cycling cells, zebrafish were immersed in 5 mM 5-bromo-2′-deoxyuridine (Sigma) solution overnight. The BrdU was dissolved in aquarium water. Fish were immersed in groups up to 16 individuals, at a 50 ml/fish ratio. The following days after immersion, they were successively rinsed with system water and reincorporated to the fish facility.

**EdU labeling.** To label cycling cells, fish were anaesthetised in 0.01% tricaine methanosulfonate (MS222, Sigma) and a single 5-ethynyl-2′-deoxyuridine (EdU) injection (40 μL, 10 mM) was delivered intraperitoneally (i.p.). Individuals were subsequently allowed to recover in a holding tank, and returned to the fish facility.

**Brain preparation and sectioning.** After the last session, fish were deeply anaesthetised in 0.02% tricaine methanosulfonate (MS222, Sigma), brains were dissected and fixed in 4% PFA in 10 mM PBS overnight. Tissue was overprotected by immersion in 30% sucrose for 2 days, immediately frozen in liquid nitrogen and stored at −20°C. Telencephalic frontal sections of 20 μm were cut on a cryostat (Microm, HM 550), and mounted on positively charged slides. Slides were air-dried for 24 h prior to inmunofluorescence.

To analyze the pallium throughout the rostro-caudal axis, we selected four sections for each individual, designated as rostral, rostro-medial, medio-caudal and caudal. Sections were chosen according to the topological atlas Neuroanatomy of the Zebrafish Brain (Wullimann et al., 1996)⁠, see Supplementary Figure S1.

**Immunofluorescence.** Slides were rinsed three times with Tris-buffered saline (TBS) (pH = 7.4) for 5 min, incubated with 100 mM ammonium chloride for 20 min and rinsed three more times with 0.3% TritonX-100/TBS for 5 min. Then, slides were blocked with 6% bovine serum albumin (BSA), 6% normal goat serum (NGS) in TBS for 1 h and incubated overnight at 4°C with primary antibodies diluted in 6% BSA, 6% NGS in TBS. Then, they were washed with 0.3% TritonX-100/TBS four times for 5 min, and incubated with secondary antibodies coupled to Alexa Fluor 488 or 594 (1:500 dilution) for 2 h at room temperature (RT). Sections were then washed three times (5 min each) in 0.3% TritonX-100/TBS, and mounted using Fluorescence Mounting Medium (Abcam). Sections were stored at 4 °C. When double immunostaining was performed both primary, or secondary, antibodies were incubated at the same time. For BrdU and PCNA immunodetection antigen retrieval was performed before blocking: 30 min in 2 N HCl at 37°C, followed by 10 min neutralization in 0.1 M borate buffer (pH = 8,5). Primary antibodies used were: rat monoclonal anti-BrdU 1:500 (ab6326, Abcam), mouse monoclonal anti-PCNA 1:300 (PC10, sc-56, Santa Cruz Biotechnology, Inc.), mouse monoclonal anti-NeuN 1:500 (ab104224, Abcam), mouse monoclonal anti-NeuroD1 1:500 (ab60704, Abcam).

Secondary antibodies used were: goat polyclonal anti-Rat IgG Alexa Fluor 488 (ab150165, Abcam), goat polyclonal anti-Mouse IgG Alexa Fluor 488 (ab150117, Abcam), goat polyclonal anti-Rabbit IgG Alexa Fluor 594 (ab150084, Abcam), goat polyclonal anti-Mouse IgG Alexa Fluor 594 (ab150120, Abcam). The images were acquired by using an epifluorescence microscope Nikon Eclipse e800 and an ad-hoc built two-photon microscope. Colocalization of fluorescent markers were performed on single-plane images acquired in the two-photon microscope. Images were processed in FIJI (ImageJ v1.53). Immunostained sections in which the tissue was broken or folded were excluded from the analysis.

**Population dynamics model.** In this work we performed a population dynamics simulation based on the work of Than-Trong and coworkers (2020) with modifications. Briefly, the model contemplates two NSC populations, the reservoir pool (rNSCs) and the operative pool (oNSCs). Both kind of NSCs divided and differentiated following the rates reported by Than-Trong: 1) rNSC →rNSC + oNSC: (k_1_ = 0.007/day); 2) oNSC →death: (k_2_ = 0.017/day); 3) oNSC → oNSC + oNSC: (k_3_ = 0.006/day); 4) oNSC → oNSC + n: (k_4_ = 0.018/day); 5) oNSC → n + n: (k_5_ = 0.004/day); 6) oNSC → n: (k_6_ = 0.013/day). To adapt the model to our experiments, we firstly determined the system dynamics at equilibrium to establish the basal proportions of rNSC (r) and oNSC (o). Based on the reaction rates described above we developed differential equations to evaluate the temporal progression of r and o populations:

As rNSC→rNSC + oNSC: $\frac{dr}{dt}=0$ , at equilibrium r_eq_ = r(t_0_)

Whereas $\frac{do}{dt}=k_{1}r-\left(k_{3}+k_{5}+k_{6}-k_{2}\right)o=f\left(r,o\right)$ ,

at equilibrium $\frac{do}{dt}=f\left(r_{eq},o_{eq}\right)=0$ ,

with $o_{eq}=\frac{1}{4}r_{eq}$

Once established the contribution of r_eq_ and o_eq_, we developed a stochastic simulation to determine the proportions of reactions associated to cellular divisions (d_i_), which will be used as target for “BrdU labeling” simulation (Figure 6C). Hence, from this simulation we established the following parameters: d_1_ = 0.5, d_3_ = 0.11, d_4_ = 0.32, d_5_ = 0.07.

According to this, given an initial population (P_0_) of “BrdU-labeled” NSCs we established the progeny fate after a single division:

$$r_{\left(t=0\right)}=P_{0}d_{1}$$

$$o_{\left(t=0\right)}=P_{0}\left(d_{1}+2d_{3}+d_{4}\right)$$

$$n_{\left(t=0\right)}=P_{0}\left(d_{4}+2d_{5}\right)$$

Next, we compute the population dynamics through a 30 days period (see Figure 6C).

To address the way in which learning activity impinges on population dynamics, we applyed a learning factor (*λ*) to the rates of division and differentiation (k_i_) of the reactions that took place only during training periods (See Figure 6D). Based on our experiments of learning and proliferation (Figure 1 and Supplementary Figure S2), we estimated a *λ* = 3.

Neuronal death was incorporated under the hypothesis that adult-born neurons mature over time to reach a checkpoint (at day 15th), in which a portion of these neurons die with a probability p_death_ = 0.550, in accordance with the results shown in Figure 5. Next, we implemented an activity-dependent rescue to decrease neuronal death up to ten times during learning.

All the stochastic simulations were implemented in *Python* 3.8.5 using NumPy 1.19.2, and the codes are available in https://gitlab.com/n806/stochasticsimulation_learning_and_neurog.

**Statistical analysis.** Every data set was tested by the Grubbs outlier test, with alpha = 0.05. Normality was assessed using the Shapiro-Wilk test, with a p-value of 0.05. Homoscedasticity was analyzed by the Levene test, with a p-value of 0.05. When data met criteria, unpaired *t*-test or Two-way ANOVA with Bonferroni post-hoc test were used as indicated. In cases where data did not meet normality criteria, nonparametric tests used were Mann-Whitney or Kruskal–Wallis. Behavioral results were analyzed by Two-way ANOVA for repeated measures as well as by linear regressions. In all cases, statistical significance was assumed when *p* < 0.05. Unless otherwise specified, data are presented as mean ± SE. Box plots indicate median (line), 25–75% percentile (box limits), maximum and minimum values (whiskers). Simple linear regression slopes were compared with an ANCOVA test. All the statistical analyses performed in this work are detailed in the supplementary information file.

## Data Availability

The original contributions presented in the study are included in the article/Supplementary Material, further inquiries can be directed to the corresponding author.

## References

[B1] Aasebø I. E. J. Kasture A. S. Passeggeri M. Tashiro A. (2018). A Behavioral Task with More Opportunities for Memory Acquisition Promotes the Survival of New Neurons in the Adult Dentate Gyrus. Sci. Rep. 8, 1–11. 10.1038/s41598-018-25331-w 29743494 PMC5943398

[B2] Adams M. M. Kafaligonul H. (2018). Zebrafish-A Model Organism for Studying the Neurobiological Mechanisms Underlying Cognitive Brain Aging and Use of Potential Interventions. Front. Cel. Dev. Biol. 6, 1–5. 10.3389/fcell.2018.00135 PMC6221905 30443547

[B3] Adolf B. Chapouton P. Lam C. S. Topp S. Tannhäuser B. Strähle U. (2006). Conserved and Acquired Features of Adult Neurogenesis in the Zebrafish Telencephalon. Dev. Biol. 295, 278–293. 10.1016/j.ydbio.2006.03.023 16828638

[B4] Alonso M. Viollet C. Gabellec M.-M. Meas-Yedid V. Olivo-Marin J.-C. Lledo P.-M. (2006). Olfactory Discrimination Learning Increases the Survival of Adult-Born Neurons in the Olfactory Bulb. J. Neurosci. 26, 10508–10513. 10.1523/JNEUROSCI.2633-06.2006 17035535 PMC6674694

[B5] Alunni A. Bally-Cuif L. (2016). A Comparative View of Regenerative Neurogenesis in Vertebrates. Development 143, 741–753. 10.1242/dev.122796 26932669 PMC4813331

[B6] Alunni A. Hermel J.-M. Heuzé A. Bourrat F. Jamen F. Joly J.-S. (2010). Evidence for Neural Stem Cells in the Medaka Optic Tectum Proliferation Zones. Devel. Neurobio. 70, 693–713. 10.1002/dneu.20799 20506557

[B7] Alvarez D. D. Giacomini D. Yang S. M. Trinchero M. F. Temprana S. G. Büttner K. A. (2016). A Disynaptic Feedback Network Activated by Experience Promotes the Integration of New Granule Cells. Science 354, 459–465. 10.1594/PANGAEA.85856810.1126/science.aaf2156 27789840

[B8] Ampatzis K. Makantasi P. Dermon C. R. (2012). Cell Proliferation Pattern in Adult Zebrafish Forebrain Is Sexually Dimorphic. Neuroscience 226, 367–381. 10.1016/j.neuroscience.2012.09.022 23000628

[B9] Anand S. K. Mondal A. C. (2017). Cellular and Molecular Attributes of Neural Stem Cell Niches in Adult Zebrafish Brain. Devel. Neurobio. 77, 1188–1205. 10.1002/dneu10.1002/dneu.22508 28589616

[B10] Anderson M. L. Sisti H. M. Curlik D. M. Shors T. J. Curlik D. M. Shors T. J. (2011). Associative Learning Increases Adult Neurogenesis During a Critical Period. Eur. J. Neurosci. 33, 175–181. 10.1111/j.1460-9568.2010.07486.x 21143670 PMC3057912

[B11] Ausas M. S. Mazzitelli-Fuentes L. Roman F. R. Crichigno S. A. De Vincenti A. P. Mongiat L. A. (2019). Social Isolation Impairs Active Avoidance Performance and Decreases Neurogenesis in the Dorsomedial Telencephalon of Rainbow Trout. Physiol. Behav. 198, 1–10. 10.1016/j.physbeh.2018.10.006 30296403

[B12] Barnea A. Nottebohm F. Barnea A. Nottenbhom F. (1994). Seasonal Recruitment of Hippocampal Neurons in Adult Free-Ranging Black-Capped Chickadees. Proc. Natl. Acad. Sci. U.S.A. 91, 11217–11221. 10.1073/pnas.91.23.11217 7972037 PMC45198

[B13] Dirian L. Galant S. Coolen M. Chen W. Bedu S. Houart C. (2014). Spatial Regionalization and Heterochrony in the Formation of Adult Pallial Neural Stem Cells. Dev. Cel. 30, 123–136. 10.1016/j.devcel.2014.05.012 25017692

[B14] Durán E. Ocaña F. M. Broglio C. Rodríguez F. Salas C. Durán E. (2010). Lateral but Not Medial Telencephalic Pallium Ablation Impairs the Use of Goldfish Spatial Allocentric Strategies in a “Hole-Board” Task. Behav. Brain Res. 214, 480–487. 10.1016/j.bbr.2010.06.010 20600353

[B15] Elliott S. B. Harvey-Girard E. Giassi A. C. C. Maler L. (2017). Hippocampal-Like Circuitry in the Pallium of an Electric Fish: Possible Substrates for Recursive Pattern Separation and Completion. J. Comp. Neurol. 525, 8–46. 10.1002/cne.24060 27292574

[B16] Folgueira M. Bayley P. Navratilova P. Becker T. S. Wilson S. W. Clarke J. D. (2012). Morphogenesis Underlying the Development of the Everted Teleost Telencephalon. Neural Dev. 7, 32. 10.1186/1749-8104-7-32 22989074 PMC3520737

[B17] Fotowat H. Lee C. Jun J. J. Maler L. (2019). Neural Activity in a Hippocampus-Like Region of the Teleost Pallium Is Associated with Active Sensing and Navigation. Elife 8, 1–25. 10.7554/elife.44119 PMC6469930 30942169

[B18] Furlan G. Cuccioli V. Vuillemin N. Dirian L. Muntasell A. J. Coolen M. (2017). Life-Long Neurogenic Activity of Individual Neural Stem Cells and Continuous Growth Establish an Outside-In Architecture in the Teleost Pallium. Curr. Biol. 27, 3288–3301. 10.1016/j.cub.2017.09.052 29107546 PMC5678050

[B19] Ganz J. Kroehne V. Freudenreich D. Machate A. Geffarth M. Braasch I. (2015). Subdivisions of the Adult Zebrafish Pallium Based on Molecular Marker Analysis. F1000Res 3, 308–318. 10.12688/f1000research.5595.1 PMC4335597 25713698

[B20] Ge S. Yang C.-h. Hsu K.-S. Ming G.-l. Song H. (2007). A Critical Period for Enhanced Synaptic Plasticity in Newly Generated Neurons of the Adult Brain. Neuron 54, 559–566. 10.1016/j.neuron.2007.05.002 17521569 PMC2040308

[B21] Goldman S. A. Nottebohm F. (1983). Neuronal Production, Migration, and Differentiation in a Vocal Control Nucleus of the Adult Female Canary Brain. Proc. Natl. Acad. Sci. U.S.A. 80, 2390–2394. 10.1073/pnas.80.8.2390 6572982 PMC393826

[B22] Grandel H. Brand M. (2013). Comparative Aspects of Adult Neural Stem Cell Activity in Vertebrates. Dev. Genes Evol. 223, 131–147. 10.1007/s00427-012-0425-5 23179636

[B23] Grandel H. Kaslin J. Ganz J. Wenzel I. Brand M. (2006). Neural Stem Cells and Neurogenesis in the Adult Zebrafish Brain: Origin, Proliferation Dynamics, Migration and Cell Fate. Dev. Biol. 295, 263–277. 10.1016/j.ydbio.2006.03.040 16682018

[B24] Harvey-Girard E. Giassi A. C. C. Ellis W. Maler L. (2012). Organization of the Gymnotiform Fish Pallium in Relation to Learning and Memory: IV. Expression of Conserved Transcription Factors and Implications for the Evolution of Dorsal Telencephalon. J. Comp. Neurol. 520, 3395–3413. 10.1002/cne.23107 22430363

[B25] Ingle D. Sahagian D. (1973). Solution of a Spatial Constancy Problem by Goldfish. Psychobiology 1, 83–84. 10.3758/BF03326873

[B26] Iribarne L. Castelló M. E. (2014). Postnatal Brain Development of the Pulse Type, Weakly Electric Gymnotid Fish Gymnotus Omarorum. J. Physiology-Paris 108, 47–60. 10.1016/j.jphysparis.2014.05.001 24844821

[B27] Jerison H. J. (1973). Evolution of the Brain and Intelligence. 1973rd ed. New York: Elsevier. Academic Press. Available at: https://www.sciencedirect.com/book/9780123852502/evolution-of-the-brain-and-intelligence#book-description .

[B28] Kaslin J. Ganz J. Brand M. (2008). Proliferation, Neurogenesis and Regeneration in the Non-Mammalian Vertebrate Brain. Phil. Trans. R. Soc. B 363, 101–122. 10.1098/rstb.2006.2015 17282988 PMC2605489

[B29] Labusch M. Mancini L. Morizet D. Bally-Cuif L. (2020). Conserved and Divergent Features of Adult Neurogenesis in Zebrafish. Front. Cel Dev. Biol. 8, 1–28. 10.3389/fcell.2020.00525 PMC7338623 32695781

[B30] Lal P. Tanabe H. Suster M. L. Ailani D. Kotani Y. Muto A. (2018). Identification of a Neuronal Population in the Telencephalon Essential for Fear Conditioning in Zebrafish. BMC Biol. 16, 1–18. 10.1186/s12915-018-0502-y 29690872 PMC5978991

[B31] Lange C. Rost F. Machate A. Reinhardt S. Lesche M. Weber A. (2020). Single Cell Sequencing of Radial Glia Progeny Reveals Diversity of Newborn Neurons in the Adult Zebrafish Brain. Development 147, 1855951. 10.1242/dev.185595 PMC6983714 31908317

[B32] Lau B. Y. B. Mathur P. Gould G. G. Guo S. (2011). Identification of a Brain center Whose Activity Discriminates a Choice Behavior in Zebrafish. Proc. Natl. Acad. Sci. U.S.A. 108, 2581–2586. 10.1073/pnas.1018275108 21262817 PMC3038752

[B33] Leuner B. Mendolia-Loffredo S. Kozorovitskiy Y. Samburg D. Gould E. Shors T. J. (2004). Learning Enhances the Survival of New Neurons beyond the Time when the hippocampus Is Required for Memory. J. Neurosci. 24, 7477–7481. 10.1523/JNEUROSCI.0204-04.2004 15329394 PMC3279157

[B34] Lindsey B. W. Darabie A. Tropepe V. (2012). The Cellular Composition of Neurogenic Periventricular Zones in the Adult Zebrafish Forebrain. J. Comp. Neurol. 520, 2275–2316. 10.1002/cne.23065 22318736

[B35] Lindsey B. W. Di Donato S. Kaslin J. Tropepe V. (2014). Sensory-specific Modulation of Adult Neurogenesis in Sensory Structures Is Associated with the Type of Stem Cell Present in the Neurogenic Niche of the Zebrafish Brain. Eur. J. Neurosci. 40, 3591–3607. 10.1111/ejn.12729 25231569

[B36] Lledo P.-M. Alonso M. Grubb M. S. (2006). Adult Neurogenesis and Functional Plasticity in Neuronal Circuits. Nat. Rev. Neurosci. 7, 179–193. 10.1038/nrn1867 16495940

[B37] Lods M. Pacary E. Mazier W. Farrugia F. Mortessagne P. Masachs N. (2021). Adult-born Neurons Immature during Learning Are Necessary for Remote Memory Reconsolidation in Rats. Nat. Commun. 12. 10.1038/s41467-021-22069-4 PMC7979763 33741954

[B38] März M. Chapouton P. Diotel N. Vaillant C. Hesl B. Takamiya M. (2010). Heterogeneity in Progenitor Cell Subtypes in the Ventricular Zone of the Zebrafish Adult Telencephalon. Glia 58, NA. 10.1002/glia.20971 20155821

[B39] Mongiat L. A. Schinder A. F. (2011). Adult Neurogenesis and the Plasticity of the Dentate Gyrus Network. Eur. J. Neurosci. 33, 1055–1061. 10.1111/j.1460-9568.2011.07603.x 21395848

[B40] Mu Y. Lee S. W. Gage F. H. (2010). Signaling in Adult Neurogenesis. Curr. Opin. Neurobiol. 20, 416–423. 10.1016/j.conb.2010.04.010 20471243 PMC2942965

[B41] Mueller T. (2011). The Conserved Bauplan of the Teleostean Telencephalon. Brain Behav. Evol. 78, 259–260. 10.1159/000331869 22086291

[B42] Mueller T. Wullimann M. F. (2009). An Evolutionary Interpretation of Teleostean Forebrain Anatomy. Brain Behav. Evol. 74, 30–42. 10.1159/000229011 19729894

[B43] Ocaña F. M. Uceda S. Arias J. L. Salas C. Rodríguez F. Rodríguez F. (2017). Dynamics of Goldfish Subregional Hippocampal Pallium Activity Throughout Spatial Memory Formation. Brain Behav. Evol. 90, 154–170. 10.1159/000478843 28988234

[B44] Olivera-Pasilio V. Peterson D. A. CastellÃ³ M. a. E. (2014). Spatial Distribution and Cellular Composition of Adult Brain Proliferative Zones in the Teleost, Gymnotus Omarorum. Front. Neuroanat. 8, 1–19. 10.3389/fnana.2014.00088 25249943 PMC4157608

[B45] Portavella M. Torres B. Salas C. (2004). Avoidance Response in Goldfish: Emotional and Temporal Involvement of Medial and Lateral Telencephalic Pallium. J. Neurosci. 24, 2335–2342. 10.1523/JNEUROSCI.4930-03.2004 14999085 PMC6730421

[B46] Prickaerts J. Koopmans G. Blokland A. Scheepens A. (2004). Learning and Adult Neurogenesis: Survival with or without Proliferation? Neurobiol. Learn. Mem. 81, 1–11. 10.1016/j.nlm.2003.09.001 14670353

[B47] Rothenaigner I. Krecsmarik M. Hayes J. A. Bahn B. Lepier A. Fortin G. (2011). Clonal Analysis by Distinct Viral Vectors Identifies Bona Fide Neural Stem Cells in the Adult Zebrafish Telencephalon and Characterizes Their Division Properties and Fate. Development 138, 1459–1469. 10.1242/dev.058156 21367818

[B48] Ruhl T. Jonas A. Seidel N. I. Prinz N. Albayram O. Bilkei-Gorzo A. (2015). Oxidation and Cognitive Impairment in the Aging Zebrafish. Gerontology 62, 47–57. 10.1159/000433534 26183067

[B49] Ryu J. R. Hong C. J. Kim J. Y. Kim E.-K. Sun W. Yu S.-W. (2016). Control of Adult Neurogenesis by Programmed Cell Death in the Mammalian Brain. Mol. Brain 9, 43. 10.1186/s13041-016-0224-4 27098178 PMC4839132

[B50] Sailor K. A. Schinder A. F. Lledo P.-M. (2017). Adult Neurogenesis Beyond the Niche: Its Potential for Driving Brain Plasticity. Curr. Opin. Neurobiol. 42, 111–117. 10.1016/j.conb.2016.12.001 28040643

[B51] Sato Y. Yano H. Shimizu Y. Tanaka H. Ohshima T. (2017). Optic Nerve Input-Dependent Regulation of Neural Stem Cell Proliferation in the Optic Tectum of Adult Zebrafish. Devel. Neurobio. 77, 474–482. 10.1002/dneu.22423 27480480

[B52] Tashiro A. Makino H. Gage F. H. (2007). Experience-Specific Functional Modification of the Dentate Gyrus Through Adult Neurogenesis: A Critical Period During an Immature Stage. J. Neurosci. 27, 3252–3259. 10.1523/JNEUROSCI.4941-06.2007 17376985 PMC6672473

[B53] Than-Trong E. Kiani B. Dray N. Ortica S. Simons B. Rulands S. (2020). Lineage Hierarchies and Stochasticity Ensure the Long-Term Maintenance of Adult Neural Stem Cells. Sci. Adv. 6, eaaz5424–15. 10.1126/sciadv.aaz5424 32426477 PMC7190328

[B54] Than-Trong E. Bally-cuif L. (2015). Radial Glia and Neural Progenitors in the Adult Zebrafish Central Nervous System. Glia 63, 1406–1428. 10.1002/glia.22856 25976648

[B55] Toda T. Gage F. H. (2018). Review: Adult Neurogenesis Contributes to Hippocampal Plasticity. Cell Tissue Res. 373, 693–709. 10.1007/s00441-017-2735-4 29185071

[B56] Trotha J. W. Vernier P. Bally‐Cuif L. von Trotha J. W. Vernier P. Bally-cuif L. (2014). Emotions and Motivated Behavior Converge on an Amygdala‐Like Structure in the Zebrafish. Eur. J. Neurosci. 40, 3302–3315. 10.1111/ejn.12692 25145867 PMC4278443

[B57] Uceda S. Ocaña F. M. Martín-Monzón I. Rodríguez-Expósito B. Durán E. Rodríguez F. (2015). Spatial Learning-Related Changes in Metabolic Brain Activity Contribute to the Delimitation of the Hippocampal Pallium in Goldfish. Behav. Brain Res. 292, 403–408. 10.1016/j.bbr.2015.06.018 26142782

[B58] Vargas J. P. López J. C. Portavella M. (2009). What Are the Functions of Fish Brain Pallium? Brain Res. Bull. 79, 436–440. 10.1016/j.brainresbull.2009.05.008 19463910

[B59] Wullimann M. F. Mueller T. (2004). Teleostean and Mammalian Forebrains Contrasted: Evidence from Genes to Behavior. J. Comp. Neurol. 475, 143–162. 10.1002/cne.20183 15211457

[B60] Wullimann M. F. Rupp B. Reichert H. (1996). Neuroanatomy of the Zebrafish Brain: A Topological Atlas. 1st ed. Basel: Birkhäuser Basel. 10.1007/978-3-0348-8979-7 Neuroanatomy of the Zebrafish Brain

[B61] Yang P. Kajiwara R. Tonoki A. Itoh M. (2018). Successive and Discrete Spaced Conditioning in Active Avoidance Learning in Young and Aged Zebrafish. Neurosci. Res. 130, 1–7. 10.1016/j.neures.2017.10.005 29037586

[B62] Zupanc G. K. H. (2021). Adult Neurogenesis in the Central Nervous System of Teleost Fish: From Stem Cells to Function and Evolution. J. Exp. Biol. 224. 10.1242/JEB.226357 33914040

[B63] Zupanc G. K. H. Hinsch K. Gage F. H. (2005). Proliferation, Migration, Neuronal Differentiation, and Long-Term Survival of New Cells in the Adult Zebrafish Brain. J. Comp. Neurol. 488, 290–319. 10.1002/cne.20571 15952170

[B64] Zupanc G. n. K. H. Horschke I. (1995). Proliferation Zones in the Brain of Adult Gymnotiform Fish: A Quantitative Mapping Study. J. Comp. Neurol. 353, 213–233. 10.1002/cne.903530205 7745132

