# Supplementary material for: Spatial Learning Promotes Adult Neurogenesis in Specific Regions of the Zebrafish Pallium
Source: Front Cell Dev Biol. 2022 May 11;10:840964. doi: 10.3389/fcell.2022.840964 (PMC9130729; doi:10.3389/fcell.2022.840964)
Supplement: Supplementary file 1 [file Presentation1.pdf]

Supplementary Material

Spatial learning promotes adult neurogenesis in delimited regions of the zebrafish pallium.

Mazzitelli-Fuentes Laura S, Román Fernanda R, Castillo Elías Julio R, Deleglise Emilia B and Mongiat Lucas A.

This section includes Supplementary Videos 1 and 2, and Supplementary Figures 1-5.

Supplementary Video 1. Training in the cue-guided maze of a Control individual. The video shows performance during the first and the last session of training (S1 and S5). For each session, trials 1-3 and 23-24 are presented. Note that glass barrier position shifts throughout trials.

Supplementary Video 2. Training in the cue-guided maze. The video shows performance of an individual during the first and the last session of training (S1 and S5). For each session, only trials 1-3 and 23-24 are presented. Glass barrier and exit position is indicated in the first trial.

Supplementary Figure 1

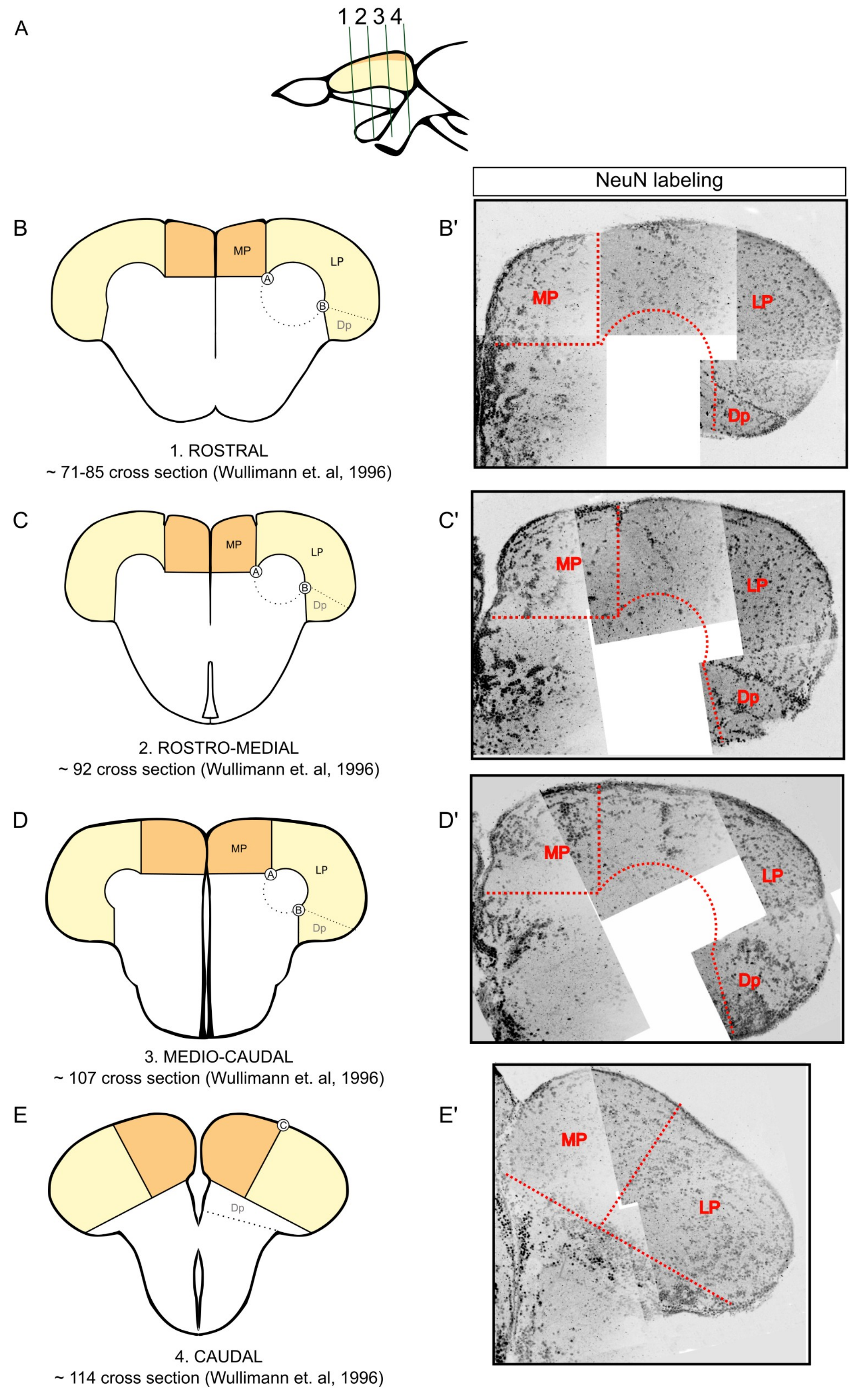

Supplementary Figure 1. A. Sagittal schematic view of zebrafish forebrain, indicating the position of rostral (1), rostromedial (2), mediocaudal (3) and caudal (4) cross sections. **B-D.** Rostral, rostromedial and mediocaudal schematic cross sections, indicating medial (MP) and lateral pallium (LP) subregions. A and B reference points delimit LP's parenchimal border. **E.** Caudal schematic cross section. C reference point delimits MP/LP border. **B'-E'.** Representative NeuN immunostained sections. Dashed line depicts MP and LP subregions. A: landmark defined as the ventro-parenchimal MP edge; B: landmark defined as the dorso-posterior pallium (Dp) parenchimal edge. C: landmark for MP border, defined as 40% of caudal pallium's total length.

Supplementary Figure 2

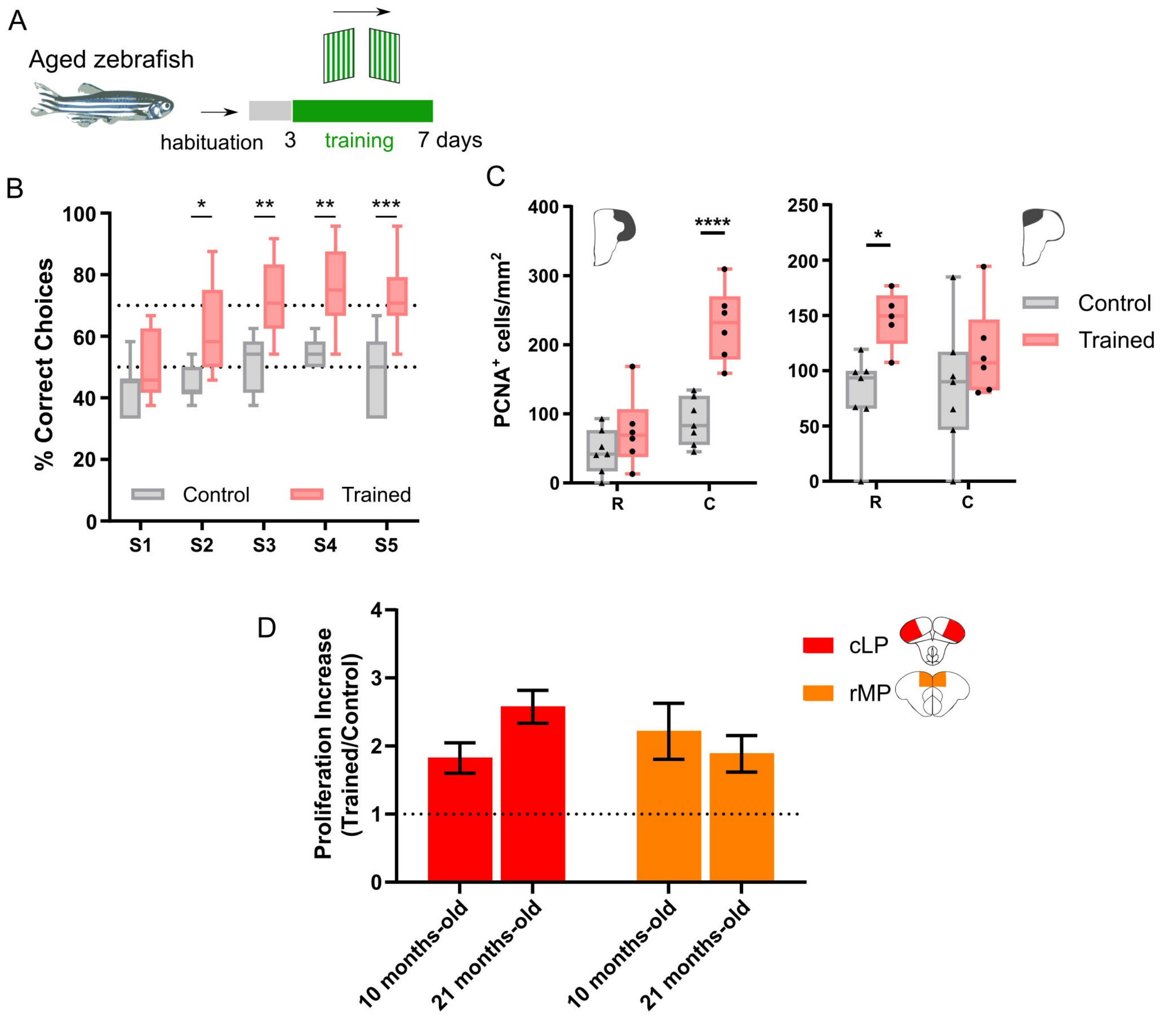

Supplementary Figure 2. A. Experimental design. Aged fish (21-months old) were subjected to a 5-session training, and euthanised immediately after for histology. **B.** Learning curves for trained and control individuals (Two-way RM ANOVA, Treatment effect: $F_{(1,\ 12)}$ = 25.68, Session effect: $F_{(4,\ 48)}$ = 7.317. Bonferroni multiple comparisons test, * p < 0.05, ** p < 0.01, *** p < 0.001). **C.** PCNA^{+} cells in LP (*left*) and MP (*right*) (Two-way ANOVA for LP: Treatment effect: $F_{(1, 22)}$ = 24,74 with p < 0.0001, Pallium region effect: $F_{(1, 22)}$ = 33.36 with p < 0.0001. Two-way ANOVA for MP: Treatment effect: $F_{(1, 21)}$ = 7.951 with p = 0.0103, Pallium region effect: $F_{(1, 21)}$ = 0.3863 with p = 0.54. Bonferroni multiple comparisons test, * p < 0.05, *** p < 0,001. Trained, n=6; Control, n=7). **D.** Learning-related proliferation ratios (Trained/Control) fron adult and aged fish are shown for comparative purposes.

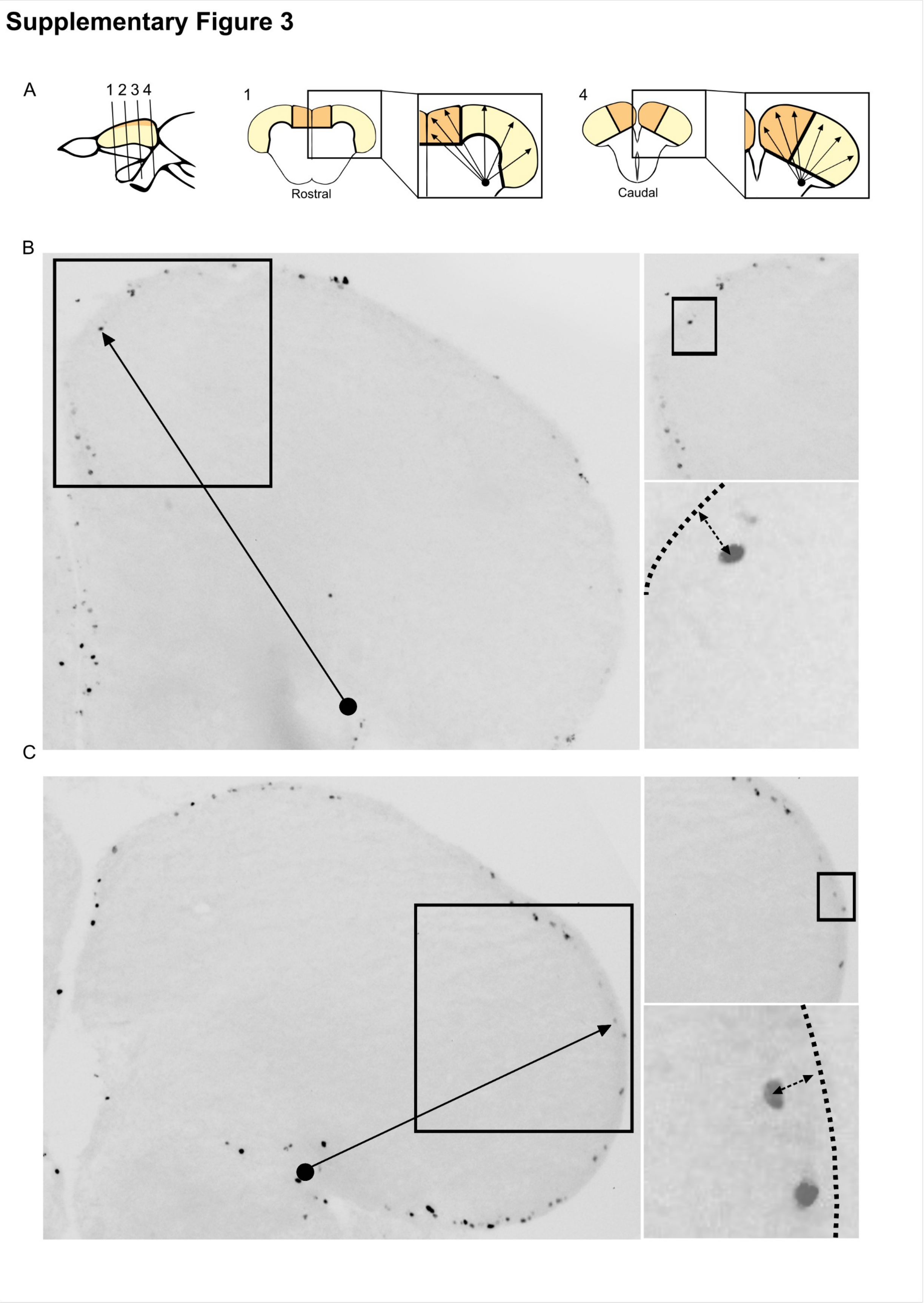

Supplementary Figure 3. A. Sagittal schematic view of zebrafish forebrain (left), indicating the position of rostral (1) and caudal (4) cross sections (right). Higher magnifications of schematical sections outline the reference point (black dot), from which virtual lines extend radially towards each BrdU^{+} cell. **B-C.** Rostral (B) and caudal (C) BrdU immunostained representative sections. Arrows depict virtual lines, linking BrdU^{+} cells and the reference point for each section. Boxed squares indicate successive magnifications. Double-headed arrows represent the migration distance from the pallium border.

Supplementary Figure 4

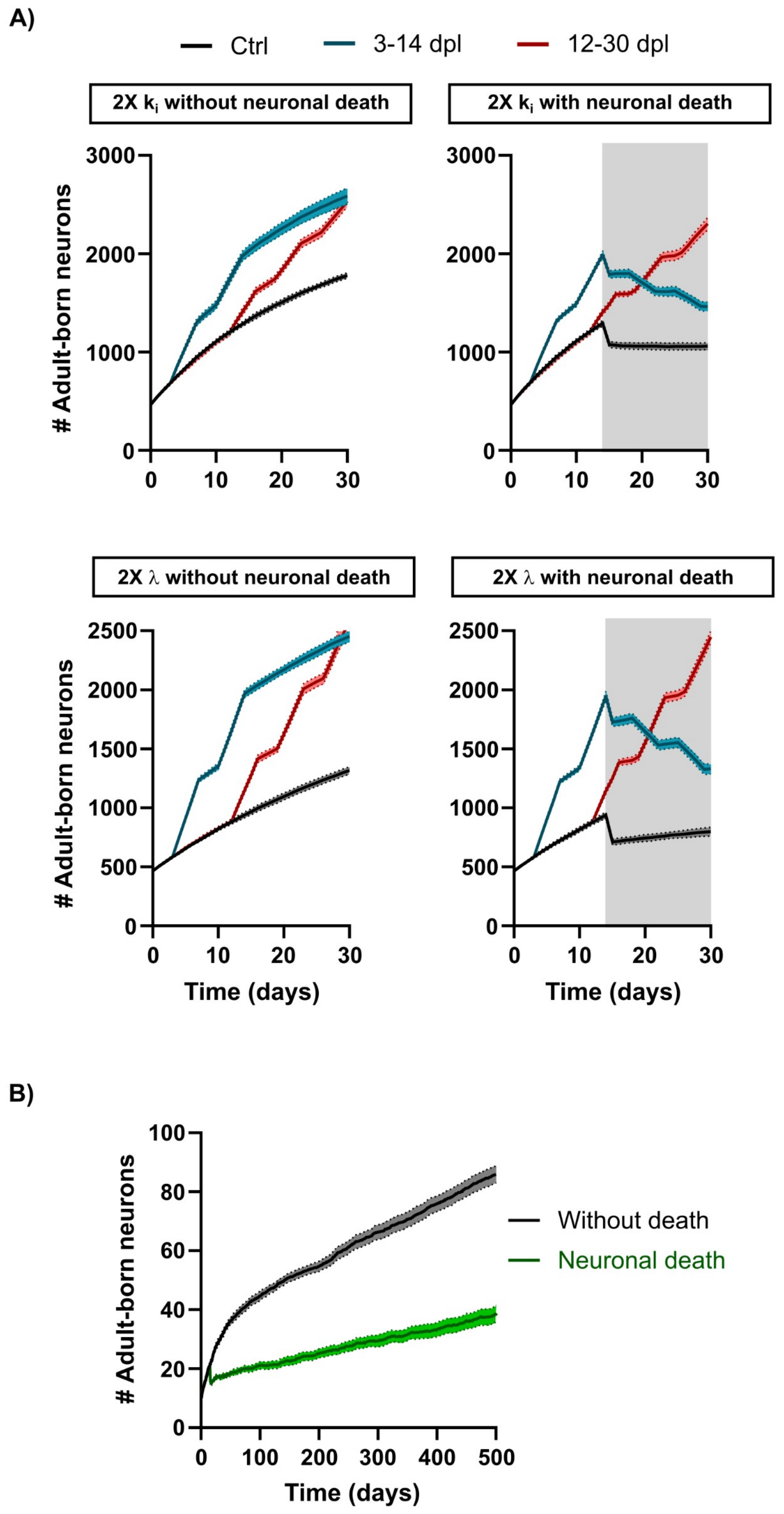

Supplementary Figure 4. A. Stochastic simulations to estimate the population of adult-born neurons in control subjects, and trained subjects during 2 training periods (3-14 dpl) and 3 training periods (12-30 dpl) under four different conditions: *Top:* 2X ki with (*right*) and without (*left*) neuronal death. *Bottom:* 2X λ with (*right*) and without (*left*) neuronal death. **B.** The plot corresponds to long-term simulations showing that adult neurogenesis in the MP is additive in both conditions, presence, and absence of neuronal death.
